# Population Pharmacokinetics, Pharmacogenomics, and Adverse Events of Osimertinib and its Two Active Metabolites, AZ5104 and AZ7550, in Japanese Patients with Advanced Non-small Cell Lung Cancer: a Prospective Observational Study

**DOI:** 10.1007/s10637-023-01328-9

**Published:** 2023-01-13

**Authors:** Emi Ishikawa, Yuta Yokoyama, Haruna Chishima, Hidefumi Kasai, Ouki Kuniyoshi, Motonori Kimura, Jun Hakamata, Hideo Nakada, Naoya Suehiro, Naoki Nakaya, Hideo Nakajima, Shinnosuke Ikemura, Ichiro Kawada, Hiroyuki Yasuda, Hideki Terai, Aya Jibiki, Hitoshi Kawazoe, Kenzo Soejima, Hiroshi Muramatsu, Sayo Suzuki, Tomonori Nakamura

**Affiliations:** 1 Division of Pharmaceutical Care Sciences, Keio University Graduate School of Pharmaceutical Sciences, Tokyo, Japan; 2 Division of Pharmaceutical Care Sciences, Center for Social Pharmacy and Pharmaceutical Care Sciences, Keio University Faculty of Pharmacy, 1-5-30 Shibakoen, Minato-ku, 105-8512 Tokyo, Japan; 3 Laboratory of Pharmacometrics and Systems Pharmacology, Keio Frontier Research and Education Collaboration Square (K-FRECS) at Tonomachi, Keio University, Kawasaki, Kanagawa Japan; 4 Department of Pharmacy, Ageo Central General Hospital, Ageo, Japan; 5 Department of Pharmacy, Keio University Hospital, Tokyo, Japan; 6 Department of Oncology, Ageo Central General Hospital, Ageo, Japan; 7 Department of Respiratory Medicine, Graduate School of Medicine, University of Yamanashi, Yamanashi, Japan; 8 Division of Pulmonary Medicine, Department of Medicine, Keio University School of Medicine, Tokyo, Japan; 9 Health Center, Keio University, Yokohama, Japan; 10 Keio Cancer Center, School of Medicine, Keio University School of Medicine, Tokyo, Japan

**Keywords:** Osimertinib, Active metabolites, Adverse events, Population pharmacokinetics, Pharmacogenomics, Therapeutic drug monitoring

## Abstract

**Background: **Potential novel strategies for adverse event (AE) management of osimertinib therapy, including therapeutic drug monitoring and the use of biomarkers, have not yet been fully investigated. This study aimed to evaluate (1) the relationship between exposure to osimertinib, especially its active metabolites (AZ5104 and AZ7550), and AEs, and (2) the relationship between germline polymorphisms and AEs. **Methods:** We conducted a prospective, longitudinal observational study of 53 patients with advanced non-small cell lung cancer receiving osimertinib therapy from February 2019 to April 2022. A population pharmacokinetic model was developed to estimate the area under the serum concentration–time curve from 0 to 24 h (AUC_0–24_) of osimertinib and its metabolites. Germline polymorphisms were analyzed using TaqMan® SNP genotyping and CycleavePCR® assays. **Results:** There was a significant association between the AUC_0–24_ of AZ7550 and grade ≥ 2 paronychia (*p* = 0.043) or anorexia (*p* = 0.011) and between that of osimertinib or AZ5104 and grade ≥ 2 diarrhea (*p* = 0.026 and *p* = 0.049, respectively). Furthermore, the AUC_0–24_ of AZ5104 was significantly associated with any grade ≥ 2 AEs (*p* = 0.046). *EGFR* rs2293348 and rs4947492 were associated with severe AEs (*p* = 0.019 and *p* = 0.050, respectively), and *ABCG2* rs2231137 and *ABCB1* rs1128503 were associated with grade ≥ 2 AEs (*p* = 0.008 and *p* = 0.038, respectively). **Conclusion:** Higher exposures to osimertinib, AZ5104, and AZ7550 and polymorphisms in *EGFR*, *ABCG2*, and *ABCB1* were related to higher severity of AEs; therefore, monitoring these may be beneficial for osimertinib AE management.

**Supplementary Information:**

The online version contains supplementary material available at 10.1007/s10637-023-01328-9.

## Introduction

Osimertinib, a third-generation epidermal growth factor receptor (EGFR)-tyrosine kinase inhibitor (TKI), is a first-line treatment for patients with advanced EGFR-positive non-small cell lung cancer (NSCLC) [1]. It has an excellent efficacy and safety profile; however, over 80% and 30% of patients administered osimertinib experience grade ≥ 2 and ≥ 3 adverse events (AEs), respectively [2]. It was initially approved as a second or later-line treatment for patients with acquired *EGFR* T790M mutation, but the indication was extended to include it as a first-line treatment and adjuvant therapy after tumor resection [3–6]. Therefore, long-term administration to asymptomatic early-stage patients is expected, increasing the importance of AE management for osimertinib treatment.

Therapeutic drug monitoring (TDM) and the use of biomarkers can be novel strategies for AE management. The potential benefits of TDM in TKI treatments have been suggested [7], and osimertinib treatments may also benefit from TDM, as it has characteristics suitable for TDM: long-term administration is required [8]; a validated bioanalytical method is available [9]; high interindividual variability of exposure with coefficients of variation of 27.6–37.6% is observed [10]; and a correlation between the exposure to osimertinib parent compound and the occurrence of AEs exists [11]. Osimertinib has two active metabolites, AZ5104 and AZ7550, which may be essential compounds for TDM. AZ5104 is produced by cytochrome P450 (CYP) 3A4 through demethylation of osimertinib indole *N-*methyl, whereas AZ7550 is produced by demethylation of the osimertinib terminal amine [12]. Both active metabolites circulate at 10% exposure of the parent compound. However, AZ5104 has a 15-fold higher potency compared with the parent compound against wild-type EGFR. In contrast, AZ7550 has a similar potency but a longer half-life; therefore, higher accumulation is expected for it than for the parent compound [8, 10]. Thus, TDM of these two active metabolites may be beneficial for AE management, but the relevance of monitoring these compounds has not yet been elucidated.

Some germline polymorphisms in the target *EGFR*, breast cancer resistance protein (BCRP/*ABCG2*), drug transporter P-glycoprotein (MDR1/*ABCB1*), and metabolism-related genes (cytochrome P450 oxidoreductase, *POR*) are associated with the occurrence of AEs or pharmacokinetics (PK) of the first- and second-generation EGFR-TKIs (gefitinib, erlotinib, and afatinib) [13–20]. These polymorphisms can act as novel biomarkers to predict osimertinib AEs, since the active metabolite of osimertinib has a high potency against wild-type *EGFR*, is a substrate of ABCG2 and ABCB1, and is metabolized by cytochrome P450, which requires electron transfer via POR [8].

While there have been reports on the association between exposure to osimertinib parent compound and its efficacy/safety [11, 21], no studies have explored the relevance of monitoring the two active metabolites of osimertinib for AE management. Additionally, germline polymorphisms in *EGFR*, *ABCG2*, *ABCB1*, or *POR* have a significant impact on the AEs of first- and second-generation EGFR-TKIs [13–20], but those of osimertinib have not been investigated.

This study aimed to evaluate (1) the exposure–toxicity relationship and (2) the association of germline polymorphisms with osimertinib AEs to provide evidence for safe treatment and quality-of-life improvement for patients with NSCLC treated with long-term administration of osimertinib.

## Methods

### Study design and patients

This prospective, longitudinal observational study was designed and conducted at the Ageo Central General Hospital from February 2019 to July 2020 and Keio University Hospital from June 2020 to April 2022. The primary endpoint was the association of exposures to osimertinib, AZ5104, AZ7550, or germline polymorphisms with AE severity. Patients with *EGFR*-mutation-positive NSCLC aged ≥ 20 years who were orally administered osimertinib (standard dose: 80 mg tablet/day) were included in this study. The inclusion criteria did not restrict the type of *EGFR* mutation, disease or treatment history, or line of treatment. Patients who were mentally or physically incapable of providing informed consent were excluded from the study. The protocol of this study was reviewed and approved by the ethics committees of Ageo Central General Hospital (Approval No. 564), Keio University School of Medicine (Approval No. 20,200,098), and Keio University Faculty of Pharmacy (Approval No. 210,118–3 and 200,710–1), and written informed consent was obtained from all participants. The study was conducted with adherence to the Declaration of Helsinki.

### Data collection

AEs were assessed during hospital stays for 3 months or at three outpatient visits, when blood was collected at 2 months or later after the initial osimertinib administration (onset of most EGFR-TKI AEs are reported to be within 2–4 months after initial administration [22–24]). The severity of AEs (Online Resource, Table D1) was scaled according to the Common Toxicity Criteria for Adverse Effects (CTCAE) version v5.0 [25] by pharmacists and physicians.

Serum samples for PK analysis and whole peripheral blood samples for genotyping were opportunistically collected (i.e., leftovers from routine laboratory blood analysis) once every 1–2 months after commencing osimertinib therapy. Patients were asked to determine the time of drug intake. The collected serum and whole peripheral blood samples were stored at − 80 °C until analysis.

The serum concentrations of the osimertinib parent compound, AZ5104, and AZ7550 were analyzed as previously described [9]. Briefly, osimertinib, AZ5104, and AZ7550 were extracted from 100 µL serum using a protein precipitation method and analyzed simultaneously using liquid chromatography–tandem mass spectrometry.

### Genotyping

Polymorphisms in *EGFR*, *ABCG2*, *ABCB1*, and *POR* (Online Resource, Appendix A) were analyzed using TaqMan® probe-based assays (Applied Biosystems, Foster City, CA, USA), whereas the *ABCG2* polymorphism (rs2231137) was studied using the CycleavePCR® assay (TaKaRa Bio Inc., Kusatsu, Japan). The detailed genotyping method is provided in the Online Resource (Appendix A).

### Exposure–toxicity and pharmacogenomics–toxicity relationship

A population pharmacokinetic (PopPK) model was developed using Phoenix® NLME™ 8.3 software (Certara, Princeton, NJ, USA) to estimate the area under the serum concentration–time curve from 0 to 24 h (AUC_0–24_) of the osimertinib parent compound, AZ5104, and AZ7550, applied as exposure measures. The detailed method for PopPK model development is provided in the Online Resource (Appendix B).

The worst grade of AE that occurred in each patient scaled using CTCAE was used for the exposure–toxicity relationship analysis. The AUC_0–24_ was simulated using the developed PopPK model based on individual predicted concentrations at the time closest to the occurrence of AEs. For patients who did not experience AEs, a median of three simulated AUC_0–24_ from three concentration data points was applied for the analysis.

Germline polymorphism and the worst grade of AE occurring in each patient were analyzed for pharmacogenomics–toxicity relationship analysis.

### Statistical analysis

The cut-off date for data collection was April 4, 2022. Continuous variables such as AUC_0–24_ and laboratory data are presented as median (interquartile range, IQR); estimates of PopPK parameters are presented as mean (standard error, SE). Exposure–toxicity and pharmacogenomics–exposure analyses (comparison of continuous variables) were performed using Mann–Whitney *U* test. Receiver operating characteristic (ROC) curve analysis was used to evaluate the discrimination potential of AUC_0–24_ for grade ≥ 2 AEs. Significance of deviation of allele and genotype frequencies from Hardy–Weinberg equilibrium was tested, and pharmacogenomics–toxicity analysis (comparison of categorical variables) was performed using Fisher’s exact test. For the pharmacogenomics association analysis, multiple statistical analyses were performed using additive, recessive, and dominant genetic models. All *p*-values were two-sided, and statistical significance was set at *p* < 0.05. IBM® SPSS® statistics version 28.0 (SPSS, Inc., Chicago, IL, USA) was used for all the statistical analyses.

## Results

### Data collection

Patient characteristics are summarized in Table 1. A total of 302 serum samples for PK analysis, median (IQR) time after administration of 6.3 (2.0–23.6) h, and 53 whole peripheral blood samples for genotyping were collected from 53 patients. The median number of serum samples per patient was five, collected within a median of 5 months. Osimertinib safety was evaluated in 51 patients; we were unable to collect enough safety data from 2 out of 53 patients because of transfer to another hospital and withdrawal of consent. The lymphocytes for one of the patients and creatine phosphokinase for 20 patients were not tested in routine clinical laboratory blood analysis. The most prevalent grade ≥ 2 AE was skin disorders (63%, Online Resource, Table D1). Approximately 25% of the patients experienced severe AEs (grade 3 or leading to dose discontinuation). The patient characteristic significantly associated with severe AEs was the EGFR-TKI treatment line, with higher frequency of severe AEs in patients receiving therapy as first-line treatment (Online Resource, Table D2, *p* = 0.004). Other characteristics, including sex, age, ECOG PS, and somatic *EGFR* mutation, were not associated with any grade ≥ 2 or ≥ 3 AEs.

**Table 1:** Patient characteristics

Patient Characteristics	No. of patients or median	% or IQR
Sex (*n*)		
Male	21	40
Female	32	60
Age (years)	69	60–74
Body weight (kg)	55	49–64
Body surface area (m^2^)	1.55	1.48–1.67
ECOG performance status (*n*)		
0	31	58
1	11	21
2	2	4
Unknown	9	17
Somatic EGFR mutation (*n*)		
Exon 19 del	28	53
Exon 21 L858R	20	38
Exon 18	1	2
Rare mutation	2	4
Compound mutation	2	4
TKI treatment line (*n*)		
First-line	37	70
Second- or later-line	16	30
Follow-up		
Safety evaluation (*n*)	51	96
Safety evaluation (months)	3	3–4
Pharmacokinetic analysis (*n*)	53	100
Pharmacokinetic sampling (months)	5	4–6
No. of pharmacokinetic samples per patient	5	4–6
Osimertinib daily dose (*n*)		
Standard dose (80 mg/day)	47	89
Dose reduction (40 mg/day or 80 mg/2 days)	5	9
Discontinuation due to adverse events	1	2
Laboratory values		
Albumin (g/dL)	4.0	3.8–4.3
Aspartate aminotransferase (U/L)	23	19–28
Alanine aminotransferase (U/L)	17	12–23
Serum creatinine (mg/dL)	0.83	0.70–1.06
Creatine kinase^a^ (U/mL)	127	63–208
Hemoglobin (g/dL)	12.4	11.6–13.2
White blood cell (1 × 10^3^ cells/µL)	4.7	3.9–5.7
Platelet count (1 × 10^3^ cells/µL)	173	156–207
Lymphocyte count^b^ (1 × 10^3^ cells/µL)	1.1	0.9–1.3

### Development and evaluation of the PopPK model

The final PopPK parameters are listed in Table 2. Albumin was identified as a significant covariate for the clearance of the parent compound and AZ5104, whereas body weight (BW) was identified as a significant covariate for the clearance of AZ7550 (Online Resource, Table C1–C3, *p* < 0.05). The PopPK parameters were estimated using models (1), (2), and (3) for osimertinib, AZ5104, and AZ7550, respectively. The estimated values were close to the mean values calculated from the bootstrap sampling of *n* = 974 (success rate, 97.4%), *n* = 1000 (success rate, 100%), and *n* = 1000 (success rate, 100%), respectively, and all fell within the 95% percentile confidence intervals (Table 2). The detailed results for the development and evaluation of the PopPK model are provided in the Online Resource (Appendix C).

**Table 2:** Parameter estimates from the final models and 1000 bootstrap samples

Parameters	Final model		Bootstrap evaluation (*n*=974^a^, *n*=1000^b^)		
	Estimate	SE	Mean	95% confidence interval	
				Lower	Upper
**CLparent/F(L/h) =** ***θ***_***1***_ **×(ALB/4)** ^***θALB***^ **×exp(ηCL)**					
*θ*_*1*_	17.37	0.886	17.25	15.37	19.35
*θALB*	1.376	0.410	1.441	0.708	2.546
Vparent/F (L)=*θ*_*2*_, K_a_ (h^-1^)=*θ*_*3*_					
*θ*_*2*_	1271	323.4	1386	847.5	2392
*θ*_*3*_	0.334	0.100	0.362	0.143	0.740
*ω*^*2*^CLparent/F	0.094	0.025	0.091	0.049	0.152
Cobs=C×(1+ε_*1*_)+ε_*2*_					
*σ*_*1*_	0.319	0.034	0.319	0.234	0.394
*σ*_*2*_	34.54	6.040	31.23	1.254	47.58
**CLm5/F(L/h) =** ***θ***_***4***_ **×(ALB/4)** ^***θm5ALB***^ **×exp(ηCLm5 + ηCLm5,IOV)**					
*θ*_*4*_	44.41	2.323	42.91	37.80	49.01
*θm5ALB*	1.935	0.514	1.966	1.005	3.072
Vm5/F (L)=*θ*_*5*_					
*θ*_*5*_	589.0	23.61	870.5	266.1	2996
*ω*^*2*^CLm5/F	0.123	0.030	0.120	0.060	0.188
*ω*^*2*^CLm5,IOV	0.034	0.007	0.034	0.017	0.055
Cm5obs=Cm5 × (1+ε_*3*_)+ε_*4*_					
*σ*_*3*_	0.266	0.029	0.263	0.197	0.321
*σ*_*4*_	2.170	0.373	1.988	0.439	2.845
**CLm7/F(L/h) =** ***θ***_***6***_ **×(BW/55)** ^***θBW***^ **×exp(ηCLm7 + ηCLm7,IOV)**					
*θ*_*6*_	46.2	0.999	46.0	41.5	51.8
*θBW*	1.25	0.0196	1.32	0.853	1.90
Vm7/F (L)=*θ*_*7*_					
*θ*_*7*_	2288	95.56	2392	1233	3775
*ω*^*2*^CLm7/F	0.0754	0.0048	0.0715	0.0371	0.107
*ω*^*2*^CLm7,IOV	0.0223	0.0047	0.0242	0.0118	0.0448
Cm7obs=Cm7 × (1+ε_*5*_)+ε_*6*_					
*σ*_*5*_	0.175	0.00524	0.173	0.123	0.216
*σ*_*6*_	2.01	0.196	1.91	0.879	2.44

CLparent/F, oral clearance of parent compound; CLm5/F, clearance of AZ5104; CLm7/F, clearance of AZ7550; Vparent/F, volume of distribution of parent compound; Vm5/F, volume of distribution of AZ5104; Vm7/F, volume of distribution of AZ7550; Cobs, observed concentration of the osimertinib parent compound; Cm5obs, observed concentration of AZ5104; Cm7obs, observed concentration of AZ7550; C, predicted concentration of the osimertinib parent compound; Cm5, predicted concentration of AZ5104; Cm7, predicted concentration of AZ7550; Ka, absorption rate constant; ALB, albumin; BW, body weight; SE, standard error; η, normally distributed random variable (mean zero and variance ω2); ω2, inter-individual or inter-occasional variance; ε, intra-individual variability; σ, intra-individual standard deviation; IOV, inter-occasion variability

a 974 successful runs from 1000 bootstrap resamplings of osimertinib parent compound data

b 1000 successful runs from 1000 bootstrap resamplings of AZ5104 and AZ7550 data

### Exposure–toxicity relationship

The median (IQR) values of AUC_0–24_ at steady state (estimated using the final PopPK model) of the osimertinib parent compound, AZ5104, and AZ7550 were 4278 (3328–5589) ng/mL*h, 414 (309–584) ng/mL*h, and 367 (275–516) ng/mL*h, respectively, regardless of dosage.

**Fig. 1:**
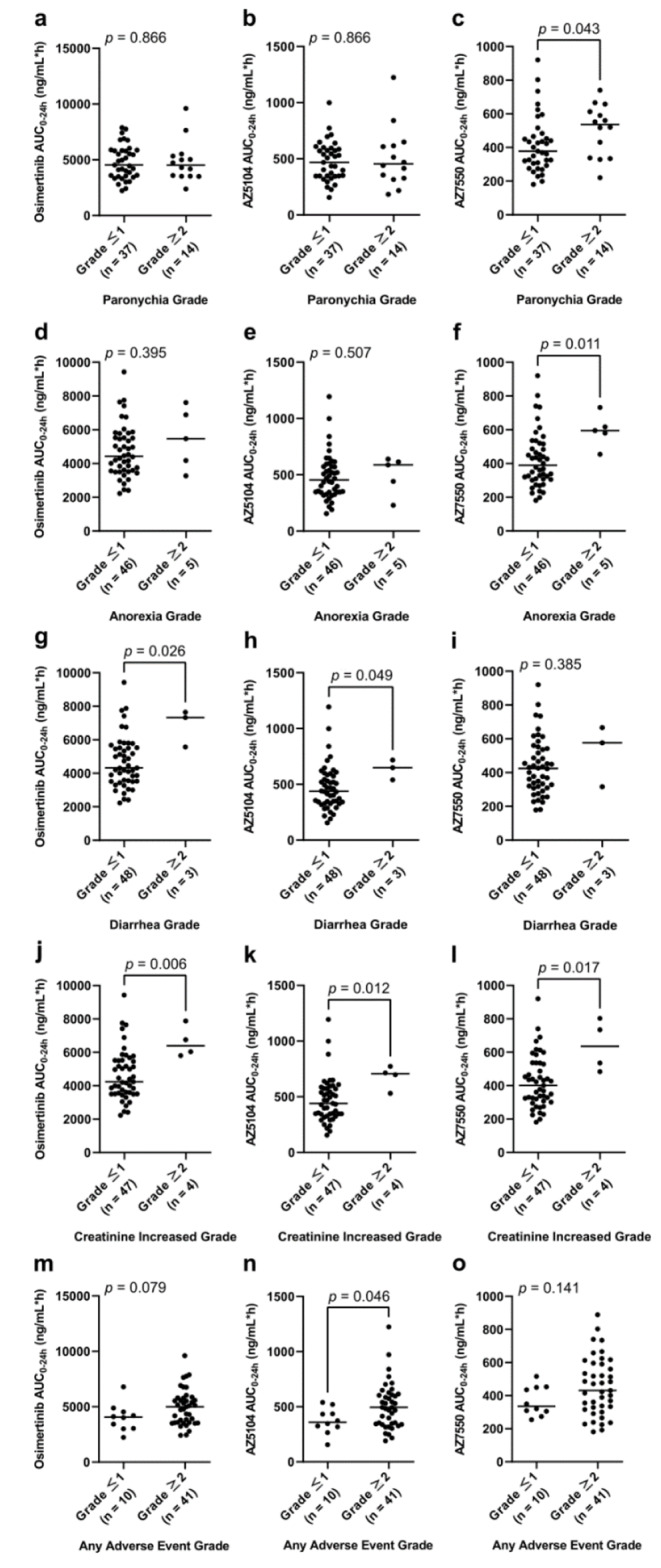
Association between the AUC_0–24_ of osimertinib parent compound and adverse events (**a**, paronychia; **d**, anorexia; **g**, diarrhea; **j**, increased creatinine; and **m**, any adverse events); association between the AUC_0–24_ of AZ5104 and adverse events (**b**, paronychia; **e**, anorexia; **h**, diarrhea; **k**, creatinine increased; and **n**, any adverse events); and association between the AUC_0–24_ of AZ7550 and adverse events (**c**, paronychia; **f**, anorexia; **i**, diarrhea; **l**, increased creatinine; and **o**, any adverse events). Solid horizontal lines indicate median values. The *p*-values are from Mann–Whitney *U* tests

There was a significant association between the AUC_0–24_ of the active metabolite AZ7550 and grade ≥ 2 paronychia or grade ≥ 2 anorexia; AUC_0–24_ of the osimertinib parent compound or active metabolite AZ5104 and grade ≥ 2 diarrhea; and AUC_0–24_ of the parent or either of the two active metabolites and grade ≥ 2 increased creatinine. Overall, the AUC_0–24_ of AZ5104 was significantly associated with any grade ≥ 2 AEs (Fig. 1).

The cut-off AUC_0–24_ of the parent compound, AZ5104, and AZ7550 to predict grade ≥ 2 AEs were 4938 ng/mL*h, 540 ng/mL*h, and 462 ng/mL*h, respectively, based on the ROC curve. The areas under the curve (sensitivity and specificity) were 0.680 (51% and 90%), 0.705 (42% and 100%), and 0.651 (49% and 90%), respectively (Online Resource, Fig. E1). The frequency of the aforementioned AEs (grade ≥ 2) was higher in patients with ≥ 4938 ng/mL*h parent compound, ≥ 540 ng/mL*h AZ5104, or ≥ 462 ng/mL*h AZ7550 AUC_0–24_ (Online Resource, Table E1–E3).

### Pharmacogenomics-toxicity relationship

All analyzed genotypes were in Hardy–Weinberg equilibrium (*p* > 0.050), except for *EGFR* rs4947492, *EGFR* rs2227983, *ABCG2* rs2622604, and *ABCB1* rs1045642 (*p* = 0.047, *p* = 0.020, *p* = 0.049, and *p* = 0.028, respectively), but the allele frequency was similar to that reported by Togo Var [26].

There was a significant relationship between polymorphisms in germline *EGFR* and severe AEs or anorexia. *EGFR* rs2293348 C > T (C/T genotype) and *EGFR* rs4947492 G > A (A/A genotype) were both associated with any severe AEs (Table 3, *p* = 0.019 in the additive model, and *p* = 0.050 in the recessive model). *EGFR* rs2293348 C > T (C/T genotype) was also significantly associated with grade ≥ 1 or ≥ 2 anorexia (Online Resource, Table F1, *p* < 0.001 or *p* = 0.023, respectively, in an additive model).

**Table 3:** Association between germline polymorphisms and any adverse events

			Any adverse events							
			Grade (No. of patients)						*p*-value^a^	
Gene	SNP ID	Genotype	1	2	3^b^			Additive model	Recessive model	Dominant model
*ABCB1*	rs1128503	C/C	4	3	1			0.072	1.000	0.038*
		C/T	2	13	6			*0.827*	*1.000*	*0.662*
		T/T	4	12	6					
*ABCB1*	rs1045642	C/C	5	11	5			0.905	1.000	0.722
		C/T	3	11	4			*0.843*	*0.474*	*1.000*
		T/T	2	6	4					
*ABCB1*	rs2032582	A/A + A/G + G/G	5	7	4			0.325	1.000	0.253
		A/T + G/T	3	16	7			*1.000*	*1.000*	*1.000*
		T/T	2	5	2					
*ABCG2*	rs2231142	C/C	4	14	4			1.000	1.000	1.000
		A/C	5	10	8			*0.485*	*1.000*	*0.348*
		A/A	1	4	1					
*ABCG2*	rs2622604	C/C	10	21	10			0.255	1.000	0.178
		C/T	0	5	3			*0.682*	*1.000*	*0.701*
		T/T	0	2	0					
*ABCG2*	rs2231137	G/G	10	16	6			0.021*	1.000	0.008*
		G/A	0	9	7			*0.124*	*0.561*	*0.192*
		A/A	0	3	0					
*POR*	rs17685	G/G	3	12	5			0.273	0.126	0.721
		G/A	4	13	7			*0.908*	*0.662*	*1.000*
		A/A	3	3	1					
*POR*	rs1057868	C/C	2	10	5			0.460	0.353	0.463
		C/T	5	15	5			*0.756*	*0.676*	*0.738*
		T/T	3	3	3					
*EGFR*	rs2293348	C/C	9	26	8			1.000		
		C/T	1	2	5			0.019*		
*EGFR*	rs4947492	G/G	1	6	0			1.000	0.728	1.000
		G/A	4	14	4			*0.064*	0.050*	*0.169*
		A/A	5	8	9					
*EGFR*	rs11977388	T/T	6	16	8			1.000	1.000	1.000
		T/C	3	7	4			*0.816*	*0.662*	*1.000*
		C/C	1	5	1					
*EGFR*	rs2227983	G/G	1	5	1			0.623	0.499	1.000
		G/A	5	9	4			*0.741*	*0.523*	*0.662*
		A/A	4	14	8					
*EGFR*	rs884225	T/T	4	8	3			0.636	1.000	0.454
		T/C	4	14	9			*0.431*	*0.417*	*0.730*
		C/C	2	6	1					

^a^First row, grade ≤ 1 versus grade ≥ 2 (Fisher’s exact test); second row (italicized), grade ≤ 2 versus grade ≥ 3 (Fisher’s exact test)

^b^Includes adverse events leading to dose discontinuation

*p < 0.050, considered statistically significant (bold)

**Fig. 2:**
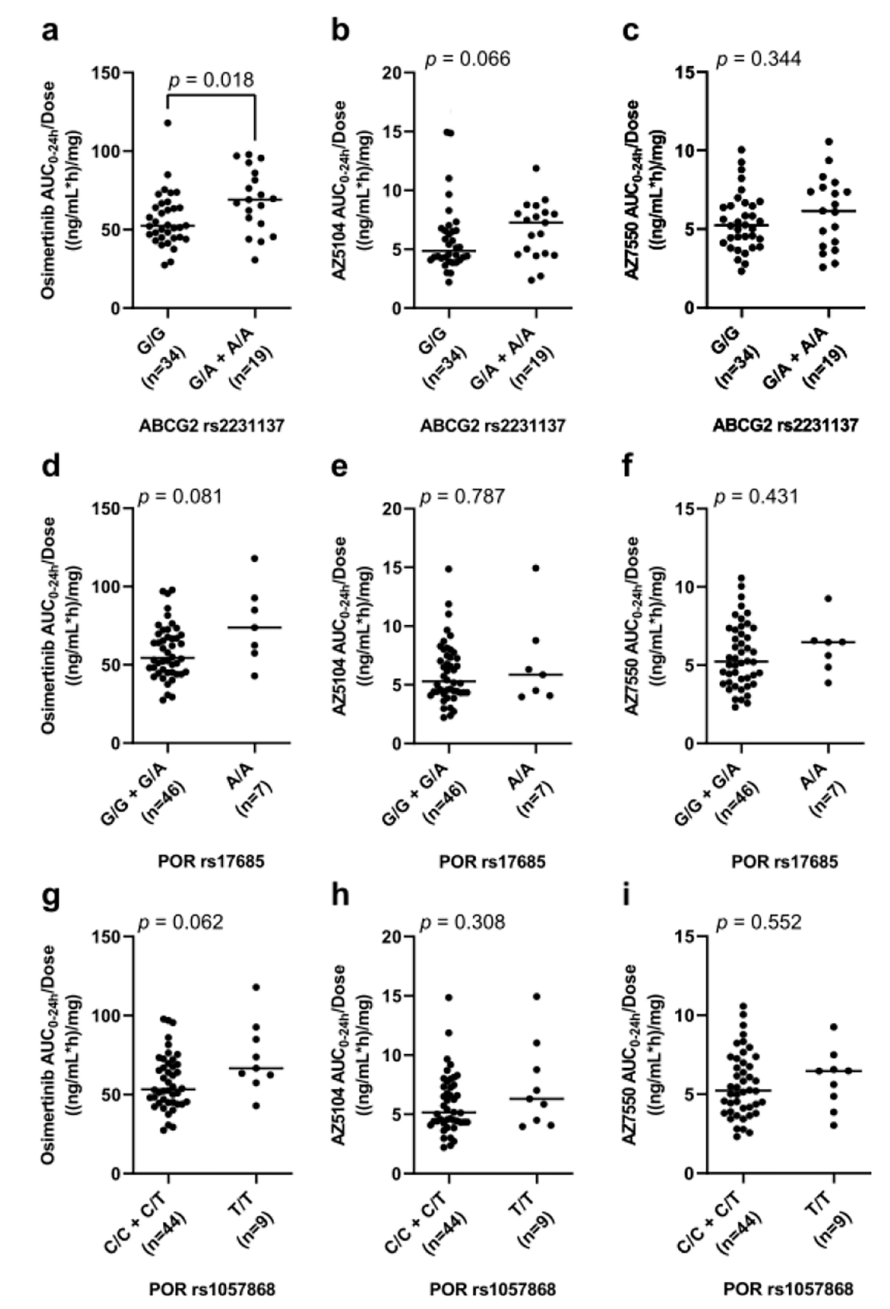
Association between the AUC_0–24_ of osimertinib parent compound and germline polymorphisms (**a**, *ABCG2* rs2231137; **d**, *POR* rs17685; and **g**, *POR* rs1057868); association between the AUC_0–24_ of AZ5104 and germline polymorphisms (**b**, *ABCG2* rs2231137; **e**, *POR* rs17685; and **h**, *POR* rs1057868); and association between the AUC_0–24_ of AZ7550 and germline polymorphisms (**c**, *ABCG2* rs2231137; **f**, *POR* rs17685; and **i**, *POR* rs1057868). Solid horizontal lines indicate median values. The *p*-values were obtained using Mann–Whitney *U* tests

The *ABCG2* rs2231137 G > A polymorphism (G/A and A/A genotypes) was significantly associated with both AEs and PK. A higher frequency of any grade ≥ 2 AEs (Table 3, *p* = 0.008 in a dominant model) was observed in patients with *ABCG2* rs2231137 G/A or A/A genotypes. Furthermore, patients with the G/A or A/A genotype had a significantly higher exposure (AUC_0–24_) to the osimertinib parent compound (Fig. 2, *p* = 0.018).

A significant association was observed between *ABCB1* rs1128503 C > T polymorphism (C/T and T/T genotypes) and any grade ≥ 2 AEs (Table 3, *p* = 0.038 in a dominant model). Additionally, the *ABCB1* rs2032582 G > T/A polymorphism (A/T and G/T genotypes) was associated with any grade ≥ 2 skin disorders and grade ≥ 2 lymphocyte count decrease (Online Resource, Table F2, *p* = 0.017; Table F3, *p* = 0.033; respectively, in an additive model). Polymorphisms in the *POR* were not significantly associated with any AEs or PK of the drug.

## Discussion

To the best of our knowledge, this is the first report to evaluate the association between exposures to the two active metabolites of osimertinib (AZ5104 and AZ7550) or germline polymorphisms (*EGFR*, *ABCG2*, *ABCB1*, and *POR*) and the severity of AEs.

PopPK modeling was performed to estimate the AUC_0–24_, which was selected as an exposure measure given the suggested linear relationship between the AUC of osimertinib parent compound (at a steady state of any dosing interval) and AEs [11]. The median estimated AUC_0–24_ of osimertinib at steady state was consistent or slightly lower than that in previous reports because some patients included in our study received a reduced dose [27, 28]. Albumin level was identified as a significant covariate for the clearance of the parent compound and AZ5104 (positive correlation), consistent with a previous report [29]. C-reactive protein (CRP) is a covariate of osimertinib clearance [21]. Decrease in albumin level and an increase in CRP level occur during inflammation, which can decrease CYP3A4 activity [30–32]. Therefore, decreased albumin level may be an indicator of increased exposures to the osimertinib parent compound and AZ5104—a consequence of decreased CYP3A4 activity during inflammation. However, elucidating these mechanisms is outside the scope of this study.

To our knowledge, our study is the first to report a PopPK model and a significant covariate (BW) for AZ7550. The BW may have an impact on the clearance of AZ7550 because AZ7550 has a longer half-life than the parent compound and AZ5104 (72.7 h versus 59.7 and 52.6 h, respectively), and greater distribution and accumulation are expected [10].

The exposure–toxicity relationships were different among the three compounds: osimertinib parent, AZ5104, and AZ7550. Exposure to AZ7550 was associated with grade ≥ 2 paronychia and anorexia, both of which are potentially related to direct epidermal or mucosal damage. The mechanisms underlying EGFR-TKI-induced skin disorders, such as paronychia, involve decreased keratinocyte proliferation and increased keratinocyte differentiation at the epidermis, resulting in impaired skin barrier function [33]. However, anorexia can be caused by multiple factors, and assessing causal associations is difficult. Although the mechanism responsible for EGFR-TKI-induced anorexia has not been elucidated, one hypothesis for anorexia induction is gastric mucosal injury, since inhibition of EGFR in gastric parietal cells can interfere with gastric mucosal membrane protection and repair of mucosal injury [34, 35]. Moreover, cancer cachexia should be considered a confounder of the AZ7550 exposure–anorexia relationship. The main characteristics of cancer cachexia are anorexia and inflammation, and the elevated production of proinflammatory cytokines can cause a decrease in CYP3A4 activity, resulting in alterations in AZ7550 metabolism [31]. However, because our PopPK analysis indicated that the albumin level, which may be related to inflammation, was not a significant covariate for AZ7550 clearance, we considered that inflammation was unlikely to affect the PK of AZ7550. It is more likely that the increase in exposure to AZ7550 increases direct epidermal or mucosal damage, leading to a higher severity of paronychia or anorexia.

Our results also indicated that exposures to the osimertinib parent compound and AZ5104 were associated with grade ≥ 2 diarrhea and increase in creatinine level. Similarly, a previous report suggested a linear relationship between the exposure to the parent compound and the occurrence of diarrhea [11], whereas another report suggested that exposure to the parent compound and grade ≥ 1 diarrhea were not associated [29]; thus, exposure to the parent compound may be related to higher grade (grade ≥ 2) diarrhea. The potential mechanism responsible for both diarrhea and creatinine increase may be the activation of chloride secretion as a result of EGFR inhibition [36]. The increased activation of chloride secretion by increased parent compound and AZ5104 exposures can enhance passive water movement through the gastrointestinal lumen, causing high-grade diarrhea and dehydration, which can lead to renal failure [36–38]. Assessment of the causal association for the exposure–kidney failure relationship is difficult because kidney failure can influence drug excretion. On the one hand, only 1.7% of the dose is excreted in the urine as osimertinib, AZ5104, and AZ7550 [39] (oral bioavailability of osimertinib: 69.8% [10]), and changes in renal clearance are unlikely to affect drug exposure [40]. On the other hand, because the influence of renal impairment on CYP3A enzyme function has been reported [41], the potential impact on total clearance cannot be ignored, and further studies are warranted to confirm this influence.

Overall, exposure to AZ5104 was significantly associated with any grade ≥ 2 AEs; the exposure to AZ5104 showed a higher area under the curve in ROC analysis than that to osimertinib parent compound and AZ7550. Likewise, an earlier study suggested an absence of a relationship between the trough concentration of the osimertinib parent compound and any toxicity [42]; thus, monitoring AZ5104 may be more beneficial than monitoring the parent compound for the management of any AEs. Additionally, the results of ROC analysis predicting any grade ≥ 2 AEs using AUC_0–24_ of the parent compound, AZ5104, and AZ7550, showed low sensitivity but high specificity. Therefore, the AUC_0–24_ levels may not be an absolute index for any AE prediction, but it may become a useful index to decide whether a patient experiencing AEs needs dose reduction. Since several reports suggested that there is no relationship between exposure to osimertinib and efficacy, dose reduction up to 50% of mean exposure may be considered for patients experiencing AEs and high AUC_0–24_ levels; however, further validation is needed [11, 21, 40, 42].

The two intron variants in *EGFR*, rs2293348 C > T (C/T genotype) and rs4947492 G > A (A/A genotype), were associated with severe AEs. According to earlier studies, *EGFR* rs2293348 is related to erlotinib-induced rash, and rs4947492 is related to gefitinib-induced diarrhea [13, 19]. The role of these genetic variants has not been fully investigated; however, since the intron sequence is involved in the regulation of expression, these variants may cause alterations in EGFR expression [43]. Thus, *EGFR* rs2293348 and rs4947492 may influence sensitivity to EGFR inhibition and severity of AEs.

The germline polymorphisms in *ABCG2* rs2231137 G > A (G/A and A/A genotypes) and *ABCB1* rs1128503 C > T (C/T and T/T genotypes) were significantly associated with any grade ≥ 2 AEs, and *ABCB1* rs2032582 G > T/A polymorphism (A/T and G/T genotypes) was significantly associated with any grade ≥ 2 skin disorders. Osimertinib is a substrate of ABCG2 and ABCB1; thus, polymorphisms in these genes may influence the distribution or PK of this drug [8, 44]. *ABCG2* rs2231137 G > A (G/A and A/A genotypes) results in Val12Met substitution and is partly responsible for the functional impairment of ABCG2. The association of this variant with gefitinib-induced rash and the PK of gefitinib has been reported [18, 45], which is consistent with our results for osimertinib. The two *ABCB1* polymorphisms (rs1128503 and rs2032582) were associated with grade ≥ 2 AEs but not with the serum concentration of osimertinib. We hypothesized that *ABCB1* polymorphisms may influence the tissue distribution and accumulation of osimertinib and its active metabolites, as an in vivo study suggested the involvement of ABCB1 and ABCG2 in tissue accumulation of other TKIs [46].

Taken together, our results suggested that monitoring exposure to the parent compound, AZ5104, and AZ7550 and genotyping polymorphisms in *EGFR, ABCG2*, and *ABCB1* are potential new approaches for AE management. AEs can be effectively managed without dose interruption by adjusting the dose and increasing awareness about the need for AE management. The AEs we focused on in this study, such as paronychia, anorexia, and diarrhea, are not fatal but need to be managed to improve quality of life and encourage patients to continue osimertinib therapy for a long period.

This study has several limitations to consider when interpreting the results. Because of the small sample size, we were unable to perform a multivariable analysis to compare the impact of multiple risk factors or check for potential confounding variables. A larger sample size is also required to evaluate the relationship between fatal or severe AEs (grade ≥ 3) and exposure to osimertinib or its active metabolites. Opportunistic collection of serum samples reduced the burden on patients but led to there being fewer concentration data points around the time of peak serum concentration (T_max_) for PopPK analysis. Additional blood sampling is required to improve the accuracy of the volume of distribution estimate, but this was not essential for the purpose of this study. The exposure–efficacy relationship was not analyzed in this study because progression-free survival was not reached for many of the participants at the time of data cut-off. Although many reports have suggested that there is no relationship between osimertinib exposure and efficacy, further studies are needed to identify the optimal therapeutic window for osimertinib treatment.

## Conclusion

Our findings demonstrated for the first time that exposures to the two active metabolites of osimertinib (AZ5104 and AZ7550) were associated with AEs and may have a different impact than exposure to the osimertinib parent compound. Therefore, monitoring not only the parent compound but also the active metabolites is a potential approach for osimertinib AE management. Germline polymorphisms in *EGFR* (rs2293348 and rs4947492), *ABCG2* (rs2231137), and *ABCB1* (rs1128503 and rs2032582) were identified as potential biomarkers for predicting the severity of AEs and may help increase awareness of the importance of AE management for patients at higher risk.

## Electronic supplementary material

Below is the link to the electronic supplementary material.

Supplementary Material 1

## Data Availability

All analyzed data are shown in the published article or in the Online Resource.
