# Supplementary material for: Population Pharmacokinetics, Pharmacogenomics, and Adverse Events of Osimertinib and its Two Active Metabolites, AZ5104 and AZ7550, in Japanese Patients with Advanced Non-small Cell Lung Cancer: a Prospective Observational Study
Source: Invest New Drugs. 2023 Jan 13;41(1):122–33. doi: 10.1007/s10637-023-01328-9 (PMC10030409; doi:10.1007/s10637-023-01328-9)
Supplement: Supplementary file 1 — Supplementary Material 1 [file 10637_2023_1328_MOESM1_ESM.docx]

Investigational New Drugs

Regular research paper

**Population Pharmacokinetics, Pharmacogenomics, and Adverse Events of Osimertinib and its Two Active Metabolites, AZ5104 and AZ7550, in Japanese Patients with Advanced Non-small Cell Lung Cancer: A Prospective Observational Study**

Emi Ishikawa^a^, Yuta Yokoyama^a,b,^*, Haruna Chishima^b^, Hidefumi Kasai^c^, Ouki Kuniyoshi^d^, Motonori Kimura^e^, Jun Hakamata^e^, Hideo Nakada^e^, Naoya Suehiro^e^, Naoki Nakaya^f^, Hideo Nakajima^f^, Shinnosuke Ikemura^g^, Ichiro Kawada^h,i^, Hiroyuki Yasuda^h^, Hideki Terai^h,j^, Aya Jibiki^b^, Hitoshi Kawazoe^a,b^, Kenzo Soejima^g^, Hiroshi Muramatsu^e^, Sayo Suzuki^a,b^, Tomonori Nakamura^a,b^

^a^Division of Pharmaceutical Care Sciences, Keio University Graduate School of Pharmaceutical Sciences, Tokyo, Japan.

^b^Division of Pharmaceutical Care Sciences, Center for Social Pharmacy and Pharmaceutical Care Sciences, Keio University Faculty of Pharmacy, Tokyo, Japan.

^c^Laboratory of Pharmacometrics and Systems Pharmacology, Keio Frontier Research and Education Collaboration Square (K-FRECS) at Tonomachi, Keio University, Kawasaki, Kanagawa, Japan.

^d^Department of Pharmacy, Ageo Central General Hospital, Ageo, Japan.

^e^Department of Pharmacy, Keio University Hospital, Tokyo, Japan.

^f^Department of Oncology, Ageo Central General Hospital, Ageo, Japan.

^g^Department of Respiratory Medicine, Graduate School of Medicine, University of Yamanashi, Yamanashi, Japan.

^h^Division of Pulmonary Medicine, Department of Medicine, Keio University School of Medicine, Tokyo, Japan.

^i^Health Center, Keio University, Yokohama, Japan.

^j^Keio Cancer Center, School of Medicine, Keio University School of Medicine, Tokyo, Japan.

*Corresponding author: Yuta Yokoyama

Division of Pharmaceutical Care Sciences, Center for Social Pharmacy and Pharmaceutical Care Sciences, Keio University Faculty of Pharmacy, 1-5-30 Shibakoen, Minato-ku, Tokyo 105-8512, Japan

Phone: +81-3-5400-2639

Fax: +81-3-5400-2651

E-mail: [yokoyama-yt@pha.keio.ac.jp](mailto:yokoyama-yt@pha.keio.ac.jp)

**Online Resource**

**Appendix A**

**Genotyping: Method**

DNA was extracted from whole peripheral blood using the QIAmp DNA Blood Mini Kit (QIAGEN, Hilden, Germany). The DNA extraction was further purified by ethanol precipitation using one-tenth volume of 3 mol/L sodium acetate, 2 volumes of 99.5% ethanol for precipitation, 2.5 volumes of 70% ethanol for rinsing (all three solvents were obtained from FUJIFILM Wako Pure Chemical Corporation, Japan). DNA yield and purity were checked by measuring the absorbance at 260 nm and 280 nm using a BioSpec-nano (Shimadzu, Kyoto, Japan). The DNA template was diluted with DNase-free distilled water (Thermo Fisher Scientific, Waltham, MA, USA) to a concentration of 1 ng/μL.

Real-time polymerase chain reaction (PCR) was performed on the Bio-Rad CFX96 real-time PCR system (Bio-Rad Laboratories, Hercules, CA, USA).

The *EGFR* polymorphisms (rs2293348, rs4947492, rs11977388, rs2227983, and rs884225), *ABCG2* (rs2231142 and rs2622604), *ABCB1* (rs1128503, rs1045642, and rs2032582), and *POR* (rs17685 and rs1057868) were analyzed using Taqman® probe-based assays (Applied Biosystems, CA, USA). The probes (FAM/VIC-fluorescent) and primers for these polymorphisms were purchased from Applied Biosystems (TaqMan® SNP Genotyping Assay ID: C _ 144945_20, C _ 27876021_10, C _ 321871_10, C _ 16170352_20, and C _ 9501579_10 for *EGFR* polymorphisms; C _ 15854163_70 and C _ 9510352_10 for *ABCG2* polymorphisms; C _ 7586662_10, C _ 7586657_20, C_11711720D_40, and C_11711720C_30 for *ABCB1* polymorphisms; and C _ 8890133_30 and C _ 8890131_30 for *POR* polymorphisms). The real-time PCR reaction mix included 4.5 ng of DNA template diluted in 4.5 µL DNase‑Free distilled water, 5 µL TaqMan® Universal Master Mix II with UNG (Uracil-N-Glycosylase), and 0.5 µL TaqMan® SNP Genotyping Assay mix, including the primers and probes, in a total volume of 10 µL. The reactions were performed under the following conditions: UNG incubation at 50 °C for 2 min and polymerase activation at 95 °C for 10 min, followed by 50 cycles of 95 °C for 15 s and 60 °C for 60 s.

*ABCG2* polymorphism (rs2231137) was studied using the CycleavePCR® assay (TaKaRa Bio Inc., Kusatsu, Shiga, Japan). The primers and probes for *ABCG2* rs2231137 were designed using the CycleavePCR Assay Designer (SNPs, TaKaRa Bio Inc.): forward 5′- TGTTCTTATCACAATGGTATGG-3′; reverse 5′-TCAGTAAATGCCTTCAGGTC-3′; FAM-labeled probe 5′-(Eclipse)TTATCCCA[G]TG(FAM)-3′; and HEX-labeled probe 5′-(Eclipse)TATCCCA[A]TGT(HEX)-3′. The real-time PCR reaction mix per reaction included 4 ng of DNA template diluted in 10.5 μL DNase‑Free distilled water, 12.5 μL CycleavePCR Reaction Mix, 0.5 μL each of forward and reverse primers, and 1 μL each of FAM/HEX-labeled probes in a total volume of 26 μL. The reactions were performed under the following conditions: initial denaturation at 95 °C for 10 s, followed by 50 cycles at 95 °C for 5 s, 55 °C for 10 s, and 72 °C for 20 s.

After real-time PCR, the result of the allelic discrimination was plotted by reading the generated fluorescence using CFX Manager Software version 3.1 (Bio-Rad Laboratories). For quality control, all the polymorphisms were genotyped more than twice.

**Appendix B**

**Population pharmacokinetics modelling: Method**

Phoenix® NLME™ 8.3 software (Certara, Princeton, NJ, USA) was used to develop a population pharmacokinetic (PopPK) model using the first-order conditional method. Several candidate PopPK models with different structures were compared based on the goodness-of-fit (GOF) plots and the objective function value (OFV) expressed as minus twice log likelihood (−2LL), and the difference in OFV between the two nested models was statistically evaluated using the chi-square test (the decrease in −2LL >3.84, *p* < 0.05, was considered statistically significant). A one-compartment model was selected with first-order absorption linking the two metabolite compartments for AZ5104 and AZ7550 (Online Resource, Fig. B1). The models were developed sequentially in the following order: (1) a model for the osimertinib parent compound; (2) a model for AZ5104, where the PK parameters regarding the parent compound were fixed at the estimated values in model (1); (3) a model for AZ7550, where the PK parameters of the parent compound and AZ5104 PK were fixed at the estimated values in models (1) and (2). The estimated PK parameters for model (1) were oral clearance of the parent compound (CLparent/F) and apparent volume of distribution of the parent compound (Vparent/F), where F represents oral bioavailability; for model (2), clearance of AZ5104 (CLm5/F) and volume of distribution of AZ5104 (Vm5/F); and for model (3), clearance of AZ7550 (CLm7/F) and volume of distribution of AZ7550 (Vm7/F). The fraction of metabolite conversion for both AZ5104 and AZ7550 from the parent was fixed at 25%, as previously reported [11].

Equation (a) was chosen to model the interindividual variability (IIV) in PK parameters based on a log-normal distribution:

*P_i_* = *θ_p_* × *exp(*η_i_), (a)

where *P_i_* is the *i*th population estimate of the PK parameters, *θ_p_* is the mean value of parameter *P_i_*, and η_i_ is a normally distributed random variable (mean zero and variance ω^2^).

The residual error is described by a combined proportional and additive error model, as shown in Equation (b):

*Cobs* = *Cpred* × (1 + ε_1_) + ε_2_, (b)

where *Cobs* is the observed serum drug concentration, *Cpred* is the predicted serum drug concentration, and ε_1_ and ε_2_ are the intraindividual variabilities (proportional error component and additive error component, respectively; mean zero and variance *σ* for concentration).

Because the serum drug concentration data were collected on multiple occasions, once every 1–2 months, from each patient, inter-occasion variability (IOV) was also evaluated.

**
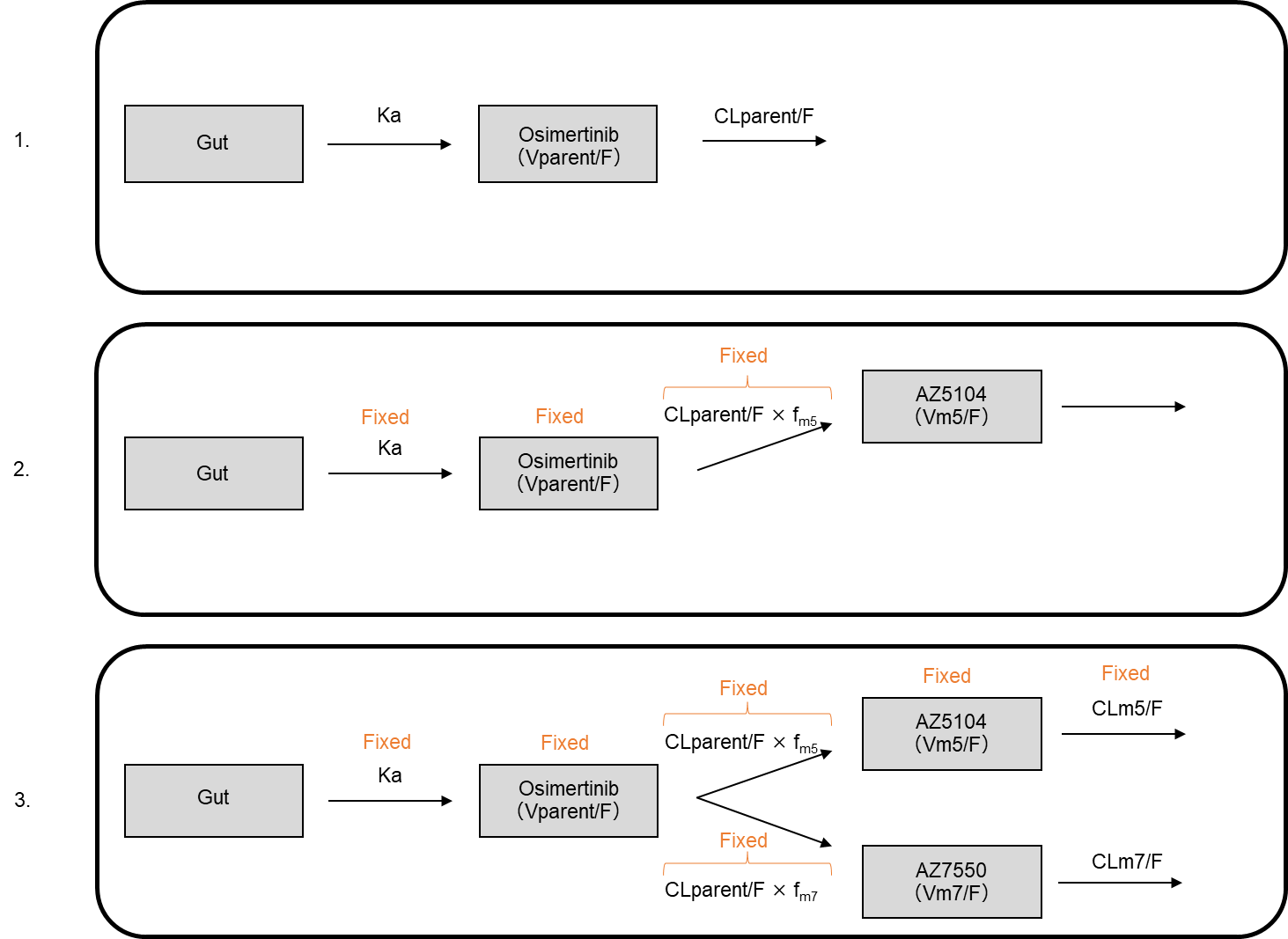
**

**Fig. B1.** Sequential development of population pharmacokinetic model. CLparent/F, oral clearance of parent compound; Ka, absorption rate constant; CLm5/F, clearance of AZ5104; CLm7/F, clearance of AZ7550; f_m5_, fraction of metabolite conversion for AZ5104; f_m7_, fraction of metabolite conversion for AZ7550; Vparent/F, volume of distribution of parent compound; Vm5/F, volume of distribution of AZ5104; and Vm7/F, volume of distribution of AZ7550.

**Covariate model: Method**

Stepwise covariate analysis was performed to evaluate the effect of covariates on clearance and volume of distribution. The significance of the effect was assessed by the −2LL value; decrease in −2LL of >3.84 (*p* < 0.050) for the forward addition was considered significant. The impact of body weight (BW), albumin (ALB), age (AGE), sex, aspartate aminotransferase (AST), and alanine aminotransferase (ALT) levels on CLparent/F, CLm5/F, and CLm7/F was evaluated. In addition, BW, ALB, AGE, and sex were evaluated on Vparent/F, Vm5/F, and Vm7/F. The covariates were modeled as shown in equation (c) in the power form for continuous covariates, equation (d) with log-transformation for continuous covariates with outliers, and equation (e) for categorical covariates:

*P_i_* = *θ_p_* × (*COV*/*median*)*^θcov^,*  (c)

*P_i_* = *θ_p_* × (1 + *log*(*COV*/*median*) × *θ_cov_*), (d)

*P_i_* = *θ_p_* × (1 + *θ_cov_* × *COV*), (e)

where *P_i_* is the *i*th population estimate of the PK parameters, *θ_p_* is the mean value of parameter *P_i_*, *COV* is the value of the covariate, *median* is the population median value of the covariates, and *θ_cov_* is the covariate coefficient.

**Model evaluation: Method**

The candidate PopPK models were evaluated by goodness of fit, ∆−2LL values, η-shrinkage, and relative standard error (RSE%). The assessed GOF plots were observed serum drug concentration (DV) versus population predictions (PRED), DV versus individual predictions (IPRED), and conditional weighted residuals (CWRES) versus PRED or time after administration (TAD). The uncertainty of the parameters was estimated by performing a bootstrap. The original dataset was randomly resampled to generate 1000 samples, and the parameters estimated from the successful runs were compared to those estimated from the original dataset.

The model was also diagnosed using a simulation-based procedure, visual predictive check (VPC), to compare the distribution or 95% percentile intervals of the predicted concentrations from the final parameters to those of the observed concentrations.

**Appendix C**

**Population pharmacokinetics modelling and covariate model: Result**

The PopPK base model was first developed without covariates using 302 serum samples; however, none of the serum drug concentration data used in this model fell below the lower limit of quantification of the analytical method. Incorporating IOV on CLm5/F and CLm7/F significantly improved the −2LL value of the base model (∆−2LL = 19.48 and ∆−2LL = 9.65, respectively), but IOV on CLparent/F did not improve the model (∆−2LL = 3.43). After base model development, a stepwise covariate analysis was performed, as shown in Online Resource, Table C1–C3. ALB on CLparent/F and AGE on CLparent/F significantly improved the −2LL value in step 1 of forward addition (∆−2LL = 13.91 and ∆−2LL = 7.44, respectively); however, because incorporating both ALB and AGE in step 2, forward addition did not significantly improve the −2LL value, only ALB on CLparent/F, showing better ∆−2LL, was added in the full version of model (1) for osimertinib parent compound analysis. As for model (2) for AZ5104, BW, ALB, AGE, and log(AST) on CLm5/F significantly decreased the −2LL value in step 1 forward addition (∆−2LL = 5.96, ∆−2LL = 27.06, ∆−2LL = 9.72, and ∆−2LL = 4.09, respectively); ALB on CLm5/F showed the highest ∆−2LL. However, ALB on CLm5/F was the only covariate added to the model because incorporating the other three covariates with ALB did not significantly improve the −2LL value. In terms of model (3) for AZ7550, BW, Male, AST, or ALT on CLm7/F improved the −2LL value in step 1 forward addition (∆−2LL = 21.35, ∆−2LL = 12.58, ∆−2LL = 5.55, and ∆−2LL = 7.74, respectively). BW on CLm7/F was the best but adding the other three covariates with BW did not significantly improve the model; thus, BW on CLm7/F was the only covariate added in the full model. The final PopPK parameters are listed in Table 2.

The IIV of Ka and volume of distribution were not estimated because η-shrinkage (standard deviation-based shrinkage) of Ka and volume of distribution were high (approximately 70% and 50%, respectively), thus, the empirical Bayes estimates were considered to be less reliable; and because the purpose of this PopPK model development was to estimate the exposure (AUC_0–24_ and clearance) to osimertinib, AZ5104, and AZ7550. The coefficients of variation for CLparent/F, CLm5/F, and CLm7/F were 30%, 35%, and 27%, respectively. The standard deviations of the additive error component and proportional error component of residual error for osimertinib parent compound, AZ5104, and AZ7550 were 34.54 ng/mL and 31.9%, 2.17 ng/mL and 26.6%, and 2.01 ng/mL and 17.5%, respectively.

**Model evaluation: Result**

GOF plots, η-shrinkage, RSE%, bootstrap, and VPC were used to evaluate the final PopPK model. The DV versus PRED and DV versus IPRED plots were relatively close to the Y=X line and symmetrically distributed (Online Resource, Fig. C1). The population prediction of observed concentrations in the higher range, i.e., concentrations around the time of peak serum concentration (T_max_), were less reliable because of the lower reliability of the mean volume of distribution estimation given the lack of observed concentration around T_max_. The CWRES versus PRED and TAD plots were distributed randomly around the CWRES=0 line (Online Resource, Fig. C2). The η-shrinkage of CLparent/F, CLm5/F, and CLm7/F was <14.4%, and the RSE% was <29.8% (data not shown). Most of the estimated PopPK parameters from models (1), (2), and (3) were close to the mean values calculated from bootstrap sampling of *n* = 974 (success rate of 97.4%), *n* = 1000 (success rate of 100%), and *n* = 1000 (success rate of 100%), respectively, and all fell within the 95% percentile confidence intervals (CIs). There was a small difference between the estimated volume of distribution (Vparent/F and Vm5/F) and the mean values obtained from the bootstrap; however, as the result showed sufficient accuracy and robustness of the clearance and the covariate coefficient estimation—the highest priority for the purpose of this study—it was considered acceptable. The VPC results showed that the distribution of the observed serum drug concentration and that of 95% CIs and 95% percentile intervals of the simulated concentration from the final PopPK parameters were relatively similar, and the model successfully described the observed serum concentration (Online Resource, Fig. C3).

**Table C1.** Summary of covariate model building of osimertinib parent compound

|  |  | Model no. |  |  |  | Model |  |  |  |  | −2LL | ∆-2(LL) |  |
| --- | --- | --- | --- | --- | --- | --- | --- | --- | --- | --- | --- | --- | --- |
|  | Base model | 1 | CLparent/F (L/h)=*θ*_1_, Vparent/F (L)=*θ*_2_, K_a_ (h^-1^)=*θ*_3_ | | | | | | | | 3472.80 |  |  |
| Forward addition | |  |  | | | | |  |  |  |  |  |  |
|  | Step 1 | 2 | Model 1 + BW on CLparent/F | | | | | | | | 3470.48 | 2.32 |  |
|  |  | 3 | Model 1 + BW on Vparent/F | | | | | | | | 3472.77 | 0.03 |  |
|  |  | 4 | Model 1 + ALB on Vparent/F | | | | | | | | 3472.89 | −0.09 |  |
|  |  | 5 | Model 1 + ALB on CLparent/F | | | | | | | | 3458.89 | 13.91 | * |
|  |  | 6 | Model 1 + AGE on CLparent/F | | | | | | | | 3465.36 | 7.44 | * |
|  |  | 7 | Model 1 + AGE on Vparent/F | | | | | | | | 3471.47 | 1.33 |  |
|  |  | 8 | Model 1 + Male on CLparent/F | | | | | | | | 3471.11 | 1.69 |  |
|  |  | 9 | Model 1 + Male on Vparent/F | | | | | | | | 3471.82 | 0.97 |  |
|  |  | 10 | Model 1 + AST on CLparent/F | | | | | | | | 3471.35 | 2.02 |  |
|  |  | 11 | Model 1 + log(AST) on CLparent/F | | | | | | | | 3470.88 | 1.92 |  |
|  |  | 12 | Model 1 + ALT on CLparent/F | | | | | | | | 3471.03 | 1.77 |  |
|  |  | 13 | Model 1 + log(ALT) on CLparent/F | | | | | | | | 3471.03 | 1.77 |  |
|  | Step 2 | 15 | Model 5 + AGE on CLparent/F | | | | | | | | 3455.09 | 3.80 |  |
|  | Full Model | 5 | Model 1 + ALB on CLparent/F | | | | | | | | 3458.89 | 13.91 |  |
| CLparent, clearance of osimertinib (parent compound); Vparent/F, volume of distribution of osimertinib (parent compound); Ka, absorption rate constant; −2LL, minus twice log likelihood; Δ(−2LL), difference in −2LL between the model and the basic model; BW, body weight; ALB, albumin; AGE, age; AST, aspartate amino transferase; ALT, alanine amino transferase.  * Statistically significant, *p* < 0.05. | | | | | | | | | | | | | |

**Table C2.** Summary of covariate model building of AZ5104

|  |  | | Model no. |  |  | Model |  |  |  |  | | |  | −2LL | Δ(−2LL) |  |
| --- | --- | --- | --- | --- | --- | --- | --- | --- | --- | --- | --- | --- | --- | --- | --- | --- |
|  | Base model | | 20 | CLm5/F (L/h)=*θ*_4_, Vm5/F (L)=*θ*_5_ | | | | | | | | | | 2008.66 |  |  |
| Forward addition | | |  |  | | | | |  | |  |  | |  |  |  |
|  | | Step 1 | 21 | Model 20 + BW on CLm5/F | | | | | | | | | | 2002.70 | 5.96 | * |
|  | |  | 22 | Model 20 + BW on Vm5/F | | | | | | | | | | 2005.73 | 2.93 |  |
|  | |  | 23 | Model 20 + ALB on Vm5/F | | | | | | | | | | 2005.96 | 2.70 |  |
|  | |  | 24 | Model 20 + ALB on CLm5/F | | | | | | | | | | 1981.60 | 27.06 | * |
|  | |  | 25 | Model 20 + AGE on CLm5/F | | | | | | | | | | 1998.94 | 9.72 | * |
|  | |  | 26 | Model 20 + AGE on Vm5/F | | | | | | | | | | 2005.47 | 3.19 |  |
|  | |  | 27 | Model 20 + Male on CLm5/F | | | | | | | | | | 2005.44 | 3.22 |  |
|  | |  | 28 | Model 20 + Male on Vm5/F | | | | | | | | | | 2005.60 | 3.06 |  |
|  | |  | 29 | Model 20 + AST on CLm5/F | | | | | | | | | | 2011.12 | −2.47 |  |
|  | |  | 30 | Model 20 + log(AST) on CLm5/F | | | | | | | | | | 2004.57 | 4.09 | * |
|  | |  | 31 | Model 20 + ALT on CLm5/F | | | | | | | | | | 2006.08 | 2.58 |  |
|  | |  | 32 | Model 20 + log(ALT) on CLm5/F | | | | | | | | | | 2005.52 | 3.14 |  |
|  | | Step 2 | 33 | Model 24 + AGE on CLm5/F | | | | | | | | | | 1979.40 | 2.20 |  |
|  | |  | 34 | Model 24 + BW on CLm5/F | | | | | | | | | | 1978.47 | 3.13 |  |
|  | |  | 35 | Model 24 + log(AST) on CLm5/F | | | | | | | | | | 1981.82 | −0.29 |  |
|  | | Full Mode | 24 | Model 20 + ALB on CLm5/F | | | | | | | | | | 1981.60 | 27.06 |  |
| CLm5/F, clearance of AZ5104; Vm5/F, volume of distribution of AZ5104; −2LL, minus twice log likelihood; Δ(−2LL), difference in −2LL between the model and the basic model; BW, body weight; ALB, albumin; AGE, age; AST, aspartate amino transferase; ALT, alanine amino transferase; f_m5_, fraction of metabolite conversion for AZ5104, fixed at 25%.  * Statistically significant, *p* < 0.05. | | | | | | | | | | | | | | | | |

**Table C3.** Summary of covariate model building of AZ7550

|  |  | Model no. |  |  | Model |  |  |  |  |  | −2LL | Δ(−2LL) |  |
| --- | --- | --- | --- | --- | --- | --- | --- | --- | --- | --- | --- | --- | --- |
|  | Base model | 40 | CLm7/F (L/h)=*θ*_6_, Vm7/F (L)=*θ*_7_ | | | | | | | | 1751.33 |  |  |
| Forward addition | |  |  | | | | |  |  |  |  |  |  |
|  | Step 1 | 41 | Model 40 + BW on CLm7/F | | | | | | | | 1729.98 | 21.35 | * |
|  |  | 42 | Model 40 + BW on Vm7/F | | | | | | | | 1748.35 | 2.98 |  |
|  |  | 43 | Model 40 + ALB on Vm7/F | | | | | | | | 1750.07 | 1.25 |  |
|  |  | 44 | Model 40 + ALB on CLm7/F | | | | | | | | 1762.96 | −11.63 |  |
|  |  | 45 | Model 40 + AGE on CLm7/F | | | | | | | | 1759.28 | −7.95 |  |
|  |  | 46 | Model 40 + AGE on Vm7/F | | | | | | | | 1750.21 | 1.11 |  |
|  |  | 47 | Model 40 + Male on CLm7/F | | | | | | | | 1738.74 | 12.58 | * |
|  |  | 48 | Model 40 + Male on Vm7/F | | | | | | | | 1759.80 | −8.47 |  |
|  |  | 49 | Model 40 + AST on CLm7/F | | | | | | | | 1745.78 | 5.55 | * |
|  |  | 50 | Model 40 + log(AST) on CLm7/F | | | | | | | | 1743.25 | 8.08 | * |
|  |  | 51 | Model 40 + ALT on CLm7/F | | | | | | | | 1743.58 | 7.74 | * |
|  |  | 52 | Model 40 + log(ALT) on CLm7/F | | | | | | | | 1746.06 | 5.27 | * |
|  | Step 2 | 53 | Model 41 + Male on CLm7/F | | | | | | | | 1729.90 | −1.92 |  |
|  |  | 54 | Model 41 + AST on CLm7/F | | | | | | | | 1730.85 | −0.87 |  |
|  |  | 55 | Model 41 + log(AST) on CLm7/F | | | | | | | | 1728.65 | 1.33 |  |
|  |  | 56 | Model 41 + ALT on CLm7/F | | | | | | | | 1727.31 | 2.67 |  |
|  |  | 57 | Model 41 + log(ALT) on CLm7/F | | | | | | | | 1726.33 | 3.65 |  |
|  | Full Model | 41 | Model 40 + BW on CLm7/F | | | | | | | | 1729.98 | 21.35 | * |
|  |  |  |  | | | | | | | |  |  |  |
| CLm7/F, clearance of AZ7550; Vm7/F, volume of distribution of AZ7550; −2LL, minus twice log likelihood; Δ(−2LL), difference in −2LL between the model and the basic model; BW, body weight; ALB, albumin; AGE, age; AST, aspartate amino transferase; ALT, alanine amino transferase; f_m7_, fraction of metabolite conversion for AZ7550, fixed at 25%.  * Statistically significant, *p* < 0.05. | | | | | | | | | | | | | |


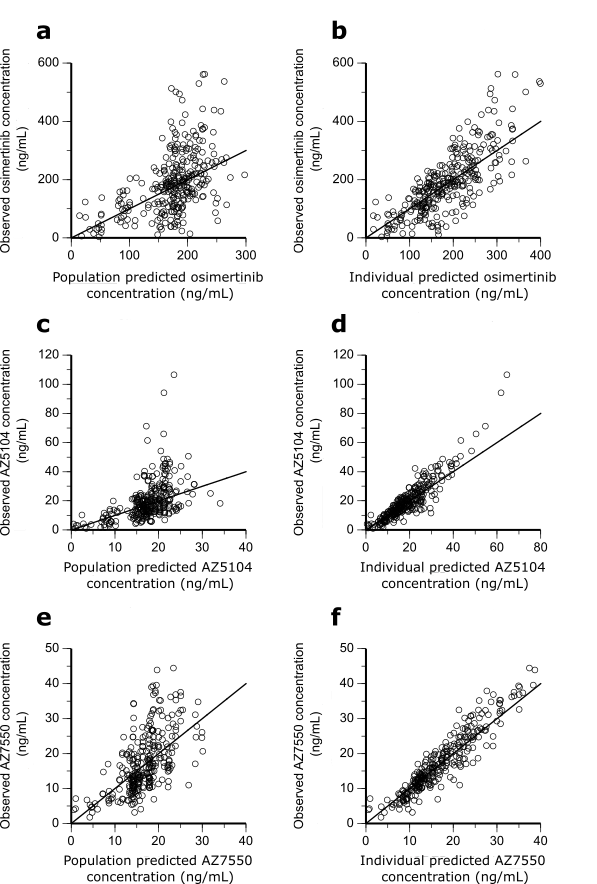


**Fig. C1.** Goodness-of-fit plots of the final population pharmacokinetics models. Observed osimertinib concentration versus population predicted osimertinib concentration, (a); observed osimertinib concentration versus individual predicted osimertinib concentration, (b); observed AZ5104 concentration versus population predicted AZ5104 concentration, (c); observed AZ5104 concentration versus individual predicted AZ5104 concentration, (d); observed AZ7550 concentration versus population predicted AZ7550 concentration, (e); and observed AZ7550 concentration versus individual predicted AZ7550 concentration, (f). Solid lines represent Y = X.


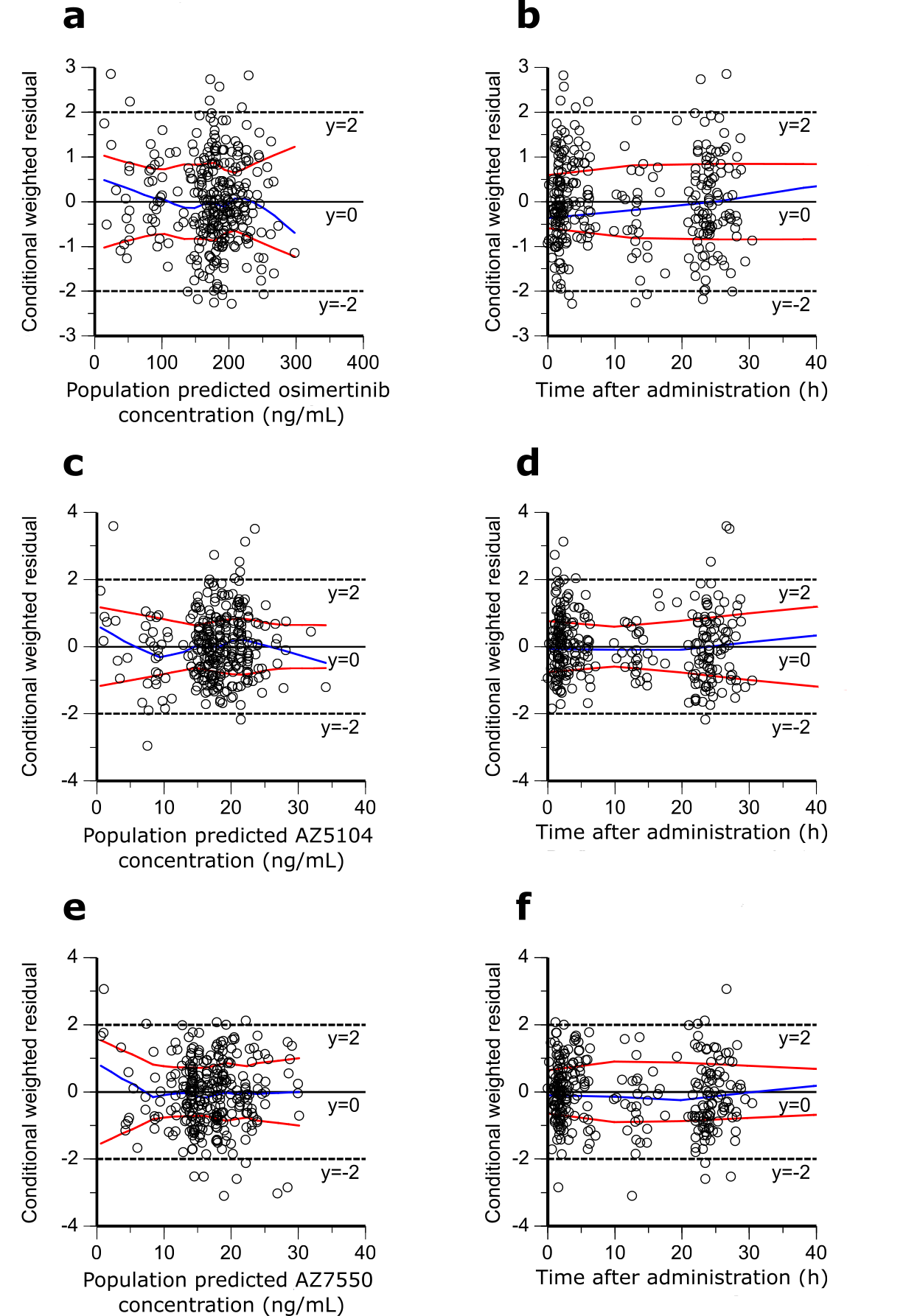


**Fig. C2.** Goodness-of-fit plots of the final population pharmacokinetics models. Conditional weighted residual versus population predicted osimertinib concentration, (a); conditional weighted residual versus time after administration for concentration of osimertinib parent compound, (b); conditional weighted residual versus population predicted AZ5104 concentration, (c); conditional weighted residual versus time after administration for concentration of AZ5104, (d); conditional weighted residual versus population predicted AZ7550 concentration, (e); and conditional weighted residual versus time after administration for concentration of AZ7550 (f). Blue lines represent locally weighted scatterplot smoothing (LOESS) regression lines, and red lines represent the LOESS regression to the absolute (or −1 times absolute) values of the dependent variable. The solid lines represent zero conditional residuals, whereas the dotted lines represent y = ±2.


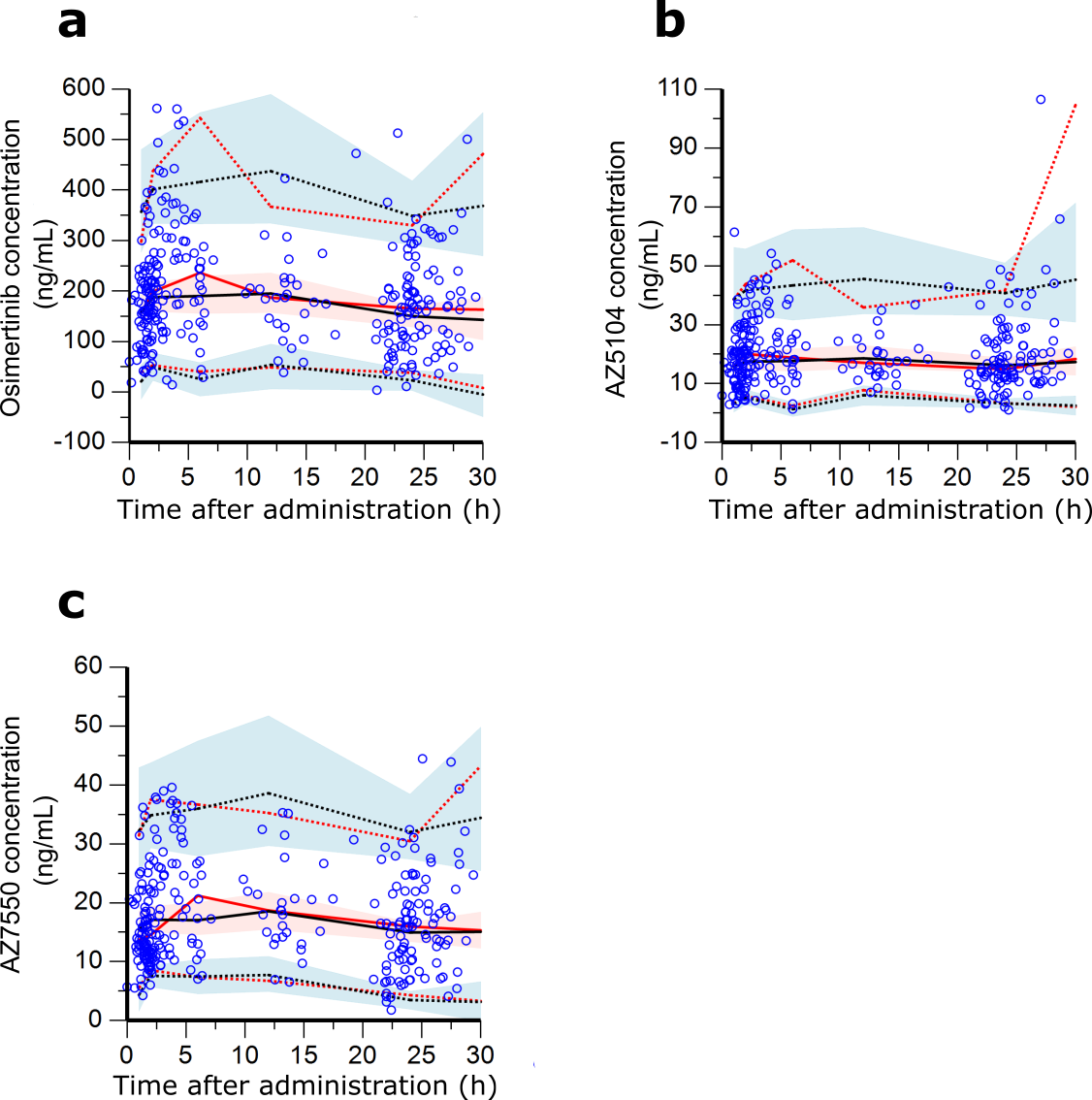


**Fig. C3.** Visual predictive checks for osimertinib parent compound concentration, (a); AZ5104 concentration, (b); and AZ7550 concentration, (c). Blue and red shades represent predicted concentration, in which each shade shows 95% percentile confidence intervals of the 5^th^, 50^th^, and 95^th^ percentiles of the predicted concentration. The blue circles are observed serum concentrations. Black solid line is the median, and black dotted lines are 5^th^ and 95^th^ percentiles of observed concentrations. Red solid line is the median, and red dotted lines are 5^th^ and 95^th^ percentiles of 1000 data sets simulated using the final population pharmacokinetic model.

**Appendix D**

**Data collection: Result**

**Table D1.** Adverse events of osimertinib

|  | No. of patients (%) Total *n* = 51 | | | | | | | | | | | | | | | | | | | | | | | | | | | | | | | | | |
| --- | --- | --- | --- | --- | --- | --- | --- | --- | --- | --- | --- | --- | --- | --- | --- | --- | --- | --- | --- | --- | --- | --- | --- | --- | --- | --- | --- | --- | --- | --- | --- | --- | --- | --- |
| Adverse Events | Any grade | | | | | grade 1 | | | | | | | | | | grade 2 | | | | | | | | grade 3^d^ | | | | | | | | |  |  |
| Skin rash | 26 |  | (51) |  | | | | 21 | |  | | (41) |  | | 4 | | |  | | | (8) | |  | | 1 | | | |  | (2) | |  | |  |
| Dry skin | 41 |  | (80) |  | | | | 18 | |  | | (35) |  | | 18 | | |  | | | (35) | |  | | 5 | | | |  | (10) | |  | |  |
| Pruritus | 31 |  | (61) |  | | | | 25 | |  | | (49) |  | | 6 | | |  | | | (12) | |  | | 0 | | | |  | (0) | |  | |  |
| Paronychia | 23 |  | (45) |  | | | | 9 | |  | | (18) |  | | 11 | | |  | | | (22) | |  | | 3 | | | |  | (6) | |  | |  |
| Any skin disorders^a^ | 48 |  | (94) |  | | | | 16 | |  | | (31) |  | | 23 | | |  | | | (45) | |  | | 9 | | | |  | (18) | |  | |  |
| Nail changes | 36 |  | (71) |  | | | | 36 | |  | | (71) |  | | 0 | | |  | | | (0) | |  | | 0 | | | |  | (0) | |  | |  |
| Diarrhea | 27 |  | (53) |  | | | | 24 | |  | | (47) |  | | 3 | | |  | | | (6) | |  | | 0 | | | |  | (0) | |  | |  |
| Constipation | 19 |  | (37) |  | | | | 12 | |  | | (24) |  | | 7 | | |  | | | (14) | |  | | 0 | | | |  | (0) | |  | |  |
| Anorexia | 7 |  | (14) |  | | | | 2 | |  | | (4) |  | | 5 | | |  | | | (10) | |  | | 0 | | | |  | (0) | |  | |  |
| Mucositis oral | 21 |  | (41) |  | | | | 18 | |  | | (35) |  | | 3 | | |  | | | (6) | |  | | 0 | | | |  | (0) | |  | |  |
| Dysgeusia | 9 |  | (18) |  | | | | 8 | |  | | (16) |  | | 1 | | |  | | | (2) | |  | | 0 | | | |  | (0) | |  | |  |
| Hair texture abnormal | 20 |  | (39) |  | | | | 20 | |  | | (39) |  | | 0 | | |  | | | (0) | |  | | 0 | | | |  | (0) | |  | |  |
| White blood cell decreased | 10 |  | (20) |  | | | | 3 | |  | | (6) |  | | 7 | | |  | | | (14) | |  | | 0 | | | |  | (0) | |  | |  |
| Platelet count decreased | 21 |  | (41) |  | | | | 20 | |  | | (39) |  | | 0 | | |  | | | (0) | |  | | 1 | | | |  | (2) | |  | |  |
| Lymphocyte count decreased^b^ | 18 |  | (36) |  | | | | 8 | |  | | (16) |  | | 8 | | |  | | | (16) | |  | | 2 | | | |  | (4) | |  | |  |
| Anemia | 27 |  | (53) |  | | | | 24 | |  | | (47) |  | | 3 | | |  | | | (6) | |  | | 0 | | | |  | (0) | |  | |  |
| Aspartate aminotransferase increased | 12 |  | (24) |  | | | | 12 | |  | | (24) |  | | 0 | | |  | | | (0) | |  | | 0 | | | |  | (0) | |  | |  |
| Alanine aminotransferase increased | 8 |  | (16) |  | | | | 8 | |  | | (16) |  | | 0 | | |  | | | (0) | |  | | 0 | | | |  | (0) | |  | |  |
| Creatinine increased | 24 |  | (47) |  | | | | 20 | |  | | (39) |  | | 4 | | |  | | | (8) | |  | | 0 | | | |  | (0) | |  | |  |
| Increased CPK^c^ | 18 |  | (58) |  | | | | 14 | |  | | (45) |  | | 1 | | |  | | | (3) | |  | | 3 | | | |  | (10) | |  | |  |
| Interstitial lung disease | 1 |  | (2) |  | | | | 0 | |  | | (0) |  | | 1 | | |  | | | (2) | |  | | 0 | | | |  | (0) | |  | |  |
| Any Adverse Events | 51 |  | (100) |  | | | | 10 | |  | | (20) |  | | 28 | | |  | | | (55) | |  | | 13 | | | |  | (25) | |  | |  |
| ^a^Any skin disorders, include skin rash, dry skin, pruritus, and paronychia. | | | | | | | | | | | | | | | | | | |  |  | |  | | | |  |  |  | | |  |  |  |  |
| ^b^ *n* = 50 (data successfully collected from 50 patients) | | |  | |  | |  | |  | |  | | |  | | |  | |  |  | |  | | | |  |  |  | | |  |  |  |  |
| ^c^ *n* = 31 (data successfully collected from 31 patients) | | | | |  | |  | |  | |  | | |  | | |  | |  |  | |  | | | |  |  |  | | |  |  |  |  |

^d^ Includes adverse events leading to dose discontinuation.

CPK, creatine phosphokinase.

**Table D2.** Association between baseline patients’ characteristics and any adverse events

|  |  | Patients without any grade ≥2 adverse events (*n* = 10) | |  | Patients with any grade ≥2 adverse events (*n* = 41) | | | | |  | *p*-value |  | | Patients without any grade ≥3^b^ adverse events (*n* = 38) | | | | | |  | Patients with any grade ≥3^b^ adverse events (*n* = 13) | | | | | | | | |  | *p*-value | | |  |  |
| --- | --- | --- | --- | --- | --- | --- | --- | --- | --- | --- | --- | --- | --- | --- | --- | --- | --- | --- | --- | --- | --- | --- | --- | --- | --- | --- | --- | --- | --- | --- | --- | --- | --- | --- | --- |
|  |  | No. of patients | % |  | No. of patients | | % | | |  |  |  | | No. of patients | | % | | | |  | No. of patients | | | | | % | | | |  |  | | |  |  |
| Sex |  |  |  |  |  | |  | | |  | 0.495 |  | |  | |  | | | |  |  | | | | |  | | | |  | 0.75 | | |  |  |
|  | Male | 3 | 30 |  | 18 | | 44 | | |  |  |  | | 15 | | 40 | | | |  | 6 | | | | | 46 | | | |  |  | | |  |  |
|  | Female | 7 | 70 |  | 23 | | 56 | | |  |  |  | | 23 | | 61 | | | |  | 7 | | | | | 54 | | | |  |  | | |  |  |
| Age (years) | |  |  |  |  | |  | | |  | 1.000 |  | |  | |  | | | |  |  | | | | |  | | | |  | 0.755 | | |  |  |
|  | <70 | 5 | 50 |  | 21 | | 51 | | |  |  |  | | 20 | | 53 | | | |  | 6 | | | | | 46 | | | |  |  | | |  |  |
|  | ≥70 | 5 | 50 |  | 20 | | 49 | | |  |  |  | | 18 | | 47 | | | |  | 7 | | | | | 54 | | | |  |  | | |  |  |
| ECOG performance status^a^ | | |  |  |  | |  | | |  | 0.756 |  | |  | |  | | | |  |  | | | | |  | | | |  | 0.219 | | |  |  |
|  | 0 | 6 | 86 |  | 25 | | 68 | | |  |  |  | | 22 | | 67 | | | |  | 9 | | | | | 82 | | | |  |  | | |  |  |
|  | 1 | 1 | 14 |  | 10 | | 27 | | |  |  |  | | 10 | | 30 | | | |  | 1 | | | | | 9 | | | |  |  | | |  |  |
|  | 2 | 0 | 0 |  | 2 | | 5 | | |  |  |  | | 1 | | 3 | | | |  | 1 | | | | | 9 | | | |  |  | | |  |  |
| Somatic *EGFR* mutation | | |  |  |  | |  | | |  | 0.477 |  | |  | |  | | | |  |  | | | | |  | | | |  | 0.086 | | |  |  |
|  | exon 19 del | 7 | 70 |  | 20 | | 49 | | |  |  |  | | 21 | | 55 | | | |  | 6 | | | | | 46 | | | |  |  | | |  |  |
|  | exon 21 L858R | 3 | 30 |  | 17 | | 42 | | |  |  |  | | 16 | | 42 | | | |  | 4 | | | | | 31 | | | |  |  | | |  |  |
|  | other | 0 | 0 |  | 4 | | 10 | | |  |  |  | | 1 | | 3 | | | |  | 3 | | | | | 23 | | | |  |  | | |  |  |
| EGFR-TKI Treatment Line | | |  |  |  | |  | | |  | 0.705 |  | |  | |  | | | |  |  | | | | |  | | | |  | **0.004*** | | |  |  |
|  | 1st | 6 | 60 |  | 29 | | 71 | | |  |  |  | | 22 | | 58 | | | |  | 13 | | | | | 100 | | | |  |  | | |  |  |
|  | 2nd or later | 4 | 40 |  | 12 | | 29 | | |  |  |  | | 16 | | 42 | | | |  | 0 | | | | | 0 | | | |  |  | | |  |  |
| ^a^ *n* = 44 (data successfully collected from 44 patients). | | | | | |  | |  |  | | | |  | |  | |  | |  | | |  | | |  | |  |  | | | |  |  |  |  |
| ^b^ Includes adverse events leading to dose discontinuation.  * *p* < 0.050, considered statistically significant (bold).  No., number; ECOG, Eastern Cooperative Oncology Group; EGFR, epidermal growth factor receptor; TKI, tyrosine kinase inhibitor. | | | | | | | | | | | | | | | | | |  | | | | |  |  | | | | |  | | | |  | |  |

**Appendix E**

**Exposure–toxicity relationship: Result**


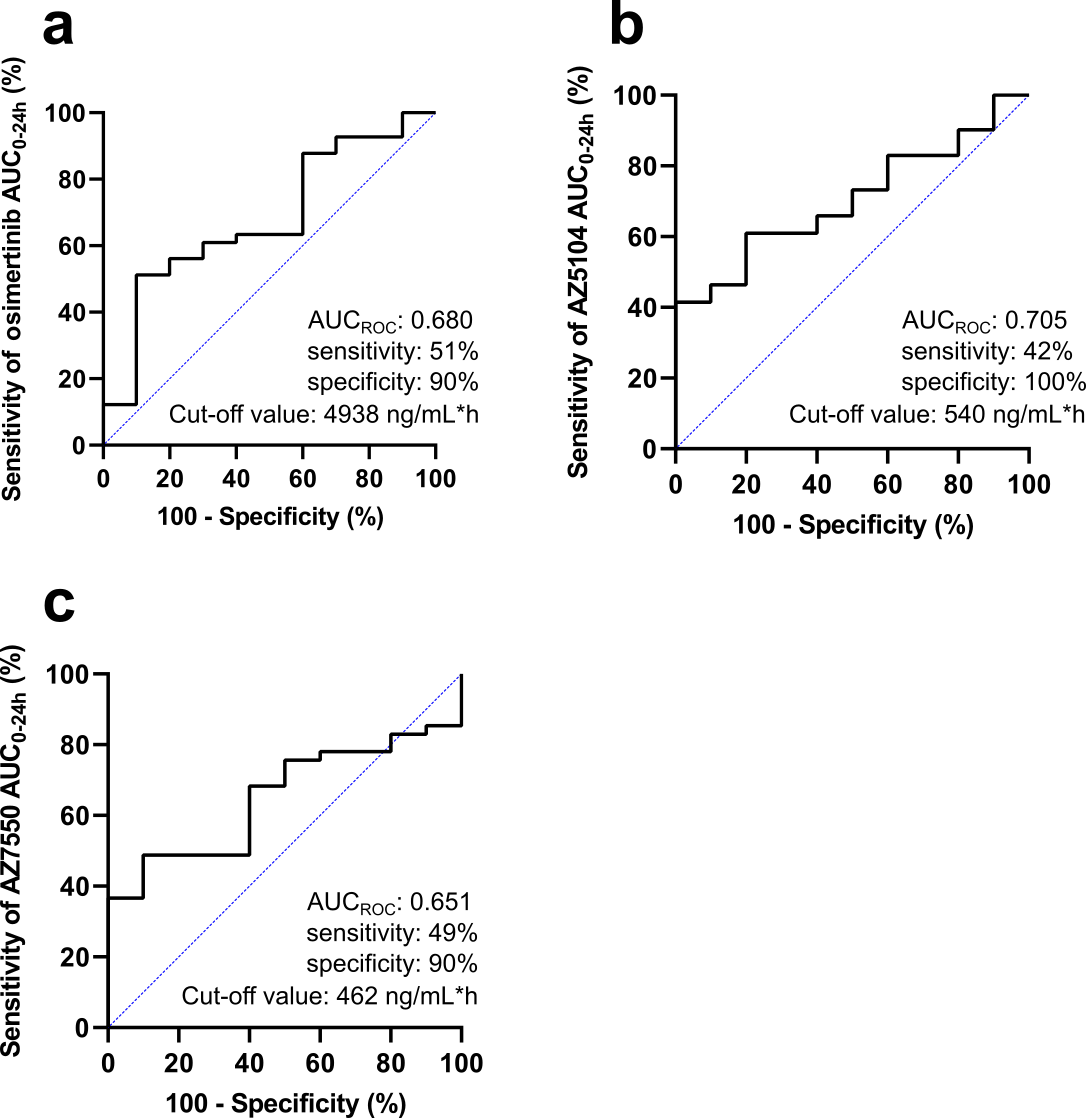


**Fig. E1.** Receiver operating characteristic (ROC) curve for predicting grade ≥2 adverse events using AUC_0-24_ of the parent compound, (a); AUC_0-24_ of AZ5104, (b); and AUC_0-24_ of AZ7550, (c).

**Table E1.** Comparison of grade ≥2 adverse events based on the osimertinib parent compound exposure

|  | Osimertinib AUC_0-24_ <4938 ng/mL*h, *n* = 29 | | | | | | |  | Osimertinib AUC_0-24_ ≥4938 ng/mL*h, *n* = 22 | | | | |
| --- | --- | --- | --- | --- | --- | --- | --- | --- | --- | --- | --- | --- | --- |
|  | Any grade | | Grade ≥2 | | |  | Any grade | | | | | Grade ≥2 | |
| Paronychia | 11 | (37.9) | | 8 | (27.6) | | |  | 12 | (54.5) | 6 | | (27.3) |
| Diarrhea | 17 | (58.6) | | 0 | (0.0) | | |  | 10 | (45.5) | 3 | | (13.6) |
| Anorexia | 3 | (10.3) | | 2 | (6.90) | | |  | 4 | (18.2) | 3 | | (13.6) |
| Creatinine increased | 13 | (44.8) | | 0 | (0.0) | | |  | 11 | (50.0) | 4 | | (18.2) |
| Interstitial lung disease | 0 | (0.0) | | 0 | (0.0) | | |  | 1 | (4.5) | 1 | | (4.5) |
| Any adverse events | 29 | (100.0) | | 20 | (69.0) | | |  | 22 | (100.0) | 21 | | (95.5) |

**Table E2.** Comparison of grade ≥ 2 adverse events based on the AZ5104 exposure

|  | AZ5104 AUC_0-24_ <540 ng/mL*h, *n* = 34 | | | | | | |  | AZ5104 AUC_0-24_ ≥540 ng/mL*h, *n* = 17 | | | | |  |
| --- | --- | --- | --- | --- | --- | --- | --- | --- | --- | --- | --- | --- | --- | --- |
|  | Any grade | | Grade ≥2 | | |  | Any grade | | | | Grade ≥2 | | | |
| Paronychia | 13 | (38.2) | | 9 | (26.5) | | |  | 10 | (58.8) | | 5 | (29.4) |  |
| Diarrhea | 19 | (55.9) | | 0 | (0.0) | | |  | 8 | (47.1) | | 3 | (17.6) |  |
| Anorexia | 4 | (11.8) | | 2 | (5.9) | | |  | 3 | (17.6) | | 3 | (17.6) |  |
| Creatinine increased | 15 | (44.1) | | 1 | (2.9) | | |  | 9 | (52.9) | | 3 | (17.6) |  |
| Interstitial lung disease | 1 | (2.9) | | 1 | (2.9) | | |  | 0 | (0.0) | | 0 | (0.0) |  |
| Any adverse events | 34 | (100.0) | | 24 | (70.6) | | |  | 17 | (100.0) | | 17 | (100.0) |  |

**Table E3.** Comparison of grade ≥ 2 adverse events based on the AZ7550 exposure

|  | AZ7550 AUC_0-24_ <462 ng/mL*h, *n* = 30 | | | | | | |  | AZ7550 AUC_0-24_ ≥462 ng/mL*h, *n* = 21 | | | | |  |
| --- | --- | --- | --- | --- | --- | --- | --- | --- | --- | --- | --- | --- | --- | --- |
|  | Any grade | | Grade ≥ 2 | | |  | Any grade | | | | Grade ≥ 2 | | | |
| Paronychia | 12 | (40.0) | | 5 | (16.7) | | |  | 11 | (52.4) | | 9 | (42.9) |  |
| Diarrhea | 19 | (63.3) | | 1 | (3.3) | | |  | 8 | (38.1) | | 2 | (9.5) |  |
| Anorexia | 3 | (10.0) | | 1 | (3.3) | | |  | 4 | (19.0) | | 4 | (19.0) |  |
| Creatinine increased | 14 | (46.7) | | 0 | (0.0) | | |  | 10 | (47.6) | | 4 | (19.0) |  |
| Interstitial lung disease | 1 | (3.3) | | 1 | (3.3) | | |  | 0 | (0.0) | | 0 | (0.0) |  |
| Any adverse events | 30 | (100.0) | | 21 | (70.0) | | |  | 21 | (100.0) | | 20 | (95.2) |  |

Grade ≥2 diarrhea and creatinine increase occurred at a higher frequency in patients with AUC_0–24_ of the parent compound ≥4938 ng/mL*h than in patients with AUC_0–24_ of the parent compound <4938 ng/mL*h; grade ≥2 diarrhea and creatinine increase occurred at a higher frequency in patients with AUC_0-24_ of AZ5104 ≥540 ng/mL*h than in patients with AUC_0–24_ of AZ5104 <540 ng/mL*h; and grade ≥2 paronychia, anorexia, and creatinine increase occurred at a higher frequency in patients with AUC_0–24_ of AZ7550 ≥462 ng/mL*h than in patients with AUC_0–24_ of AZ7550 <462 ng/mL*h (Supplementary Table E1–E3.).

No patient in our study suffered from severe liver dysfunction within the observation period. One patient out of 51 (2.0%) suffered from interstitial lung disease leading to treatment discontinuation. The patient had higher exposure to the parent compound compared to the cut-off value (5530 vs. 4938 ng/mL*h) but lower exposure to AZ5104 and AZ7550 compared to the cut-off (469 vs. 540 ng/mL*h and 423 vs. 462 ng/mL*h, respectively). This result is comparable to that of a previous study suggesting the patients who experienced pneumonitis had higher (but not significant) exposure to the parent compound (Ther Adv Med Oncol, 2022; 14:17588359221103212).

**Appendix F**

**Pharmacogenomics-toxicity relationship: Result**

**Table F1.** Association between germline polymorphisms and anorexia

|  |  |  | Anorexia | | | | | | | |
| --- | --- | --- | --- | --- | --- | --- | --- | --- | --- | --- |
|  |  |  | Grade (No. of patients) | | |  |  |  | *p*-value^a^ |  |
| Gene | SNP ID | Genotype | 0 | 1 | 2+ |  |  | Additive  model | Recessive model | Dominant  model |
| *ABCB1* | rs1128503 | C/C | 7 | 0 | 1 |  |  | 0.752 | 0.684 | 1.000 |
|  |  | C/T | 17 | 1 | 3 |  |  | *0.456* | *0.375* | *1.000* |
|  |  | T/T | 20 | 1 | 1 |  |  |  |  |  |
| *ABCB1* | rs1045642 | C/C | 19 | 1 | 1 |  |  | 0.772 | 0.662 | 0.685 |
|  |  | C/T | 15 | 1 | 2 |  |  | *0.607* | *0.580* | *0.391* |
|  |  | T/T | 10 | 0 | 2 |  |  |  |  |  |
| *ABCB1* | rs2032582 | A/A+A/G+G/G | 13 | 1 | 2 |  |  | 0.861 | 1.000 | 0.664 |
|  |  | A/T+G/T | 23 | 1 | 2 |  |  | *0.841* | *1.000* | *0.643* |
|  |  | T/T | 8 | 0 | 1 |  |  |  |  |  |
| *ABCG2* | rs2231142 | C/C | 21 | 1 | 0 |  |  | 0.199 | 1.000 | 0.124 |
|  |  | A/C | 18 | 1 | 4 |  |  | *0.097* | *0.480* | *0.062* |
|  |  | A/A | 5 | 0 | 1 |  |  |  |  |  |
| *ABCG2* | rs2622604 | C/C | 35 | 1 | 5 |  |  | 1.000 | 1.000 | 1.000 |
|  |  | C/T | 7 | 1 | 0 |  |  | *0.655* | *1.000* | *0.569* |
|  |  | T/T | 2 | 0 | 0 |  |  |  |  |  |
| *ABCG2* | rs2231137 | G/G | 29 | 1 | 2 |  |  | 0.297 | 1.000 | 0.402 |
|  |  | G/A | 12 | 1 | 3 |  |  | *0.502* | *1.000* | *0.348* |
|  |  | A/A | 3 | 0 | 0 |  |  |  |  |  |
| *POR* | rs17685 | G/G | 17 | 0 | 3 |  |  | 0.738 | 0.573 | 1.000 |
|  |  | G/A | 20 | 2 | 2 |  |  | *0.680* | *1.000* | *0.369* |
|  |  | A/A | 7 | 0 | 0 |  |  |  |  |  |
| *POR* | rs1057868 | C/C | 14 | 0 | 3 |  |  | 0.866 | 1.000 | 0.673 |
|  |  | C/T | 22 | 1 | 2 |  |  | *0.469* | *0.571* | *0.318* |
|  |  | T/T | 8 | 1 | 0 |  |  |  |  |  |
| *EGFR* | rs2293348 | C/C | 41 | 0 | 2 |  |  | **<0.001*** |  |  |
|  |  | C/T | 3 | 2 | 3 |  |  | ***0.023**** |  |  |
|  |  |  |  |  |  |  |  |  |  |  |
| *EGFR* | rs4947492 | G/G | 6 | 0 | 1 |  |  | 0.224 | 0.216 | 1.000 |
|  |  | G/A | 21 | 1 | 0 |  |  | *0.108* | *0.152* | *0.538* |
|  |  | A/A | 17 | 1 | 4 |  |  |  |  |  |
| *EGFR* | rs11977388 | T/T | 25 | 0 | 5 |  |  | 0.849 | 1.000 | 0.685 |
|  |  | T/C | 13 | 1 | 0 |  |  | *0.243* | *1.000* | *0.069* |
|  |  | C/C | 6 | 1 | 0 |  |  |  |  |  |
| *EGFR* | rs2227983 | G/G | 6 | 1 | 0 |  |  | 0.469 | 0.419 | 1.000 |
|  |  | G/A | 17 | 1 | 0 |  |  | *0.086* | *0.051* | *1.000* |
|  |  | A/A | 21 | 0 | 5 |  |  |  |  |  |
| *EGFR* | rs884225 | T/T | 13 | 0 | 2 |  |  | 1.000 | 1.000 | 1.000 |
|  |  | T/C | 23 | 2 | 2 |  |  | *0.832* | *1.000* | *0.624* |
|  |  | C/C | 8 | 0 | 1 |  |  |  |  |  |

^a^First row, grade 0 versus grade ≥1 (Fisher’s exact test); second row (italicized), grade ≤1 versus grade ≥2 (Fisher’s exact test).

** p* < 0.05, considered statistically significant (bold).

**Table F2.** Association between germline polymorphisms and any skin disorders

|  |  |  | Any skin disorders | | | | | | | |
| --- | --- | --- | --- | --- | --- | --- | --- | --- | --- | --- |
|  |  |  | Grade (No. of patients) | | |  |  |  | *p*-value^a^ |  |
| Gene | SNP ID | Genotype | 0 | 1 | 2+ |  |  | Additive  model | Recessive model | Dominant  model |
| *ABCB1* | rs1128503 | C/C | 1 | 3 | 4 |  |  | 0.545 | 1.000 | 0.407 |
|  |  | C/T | 1 | 5 | 15 |  |  | *0.499* | *0.772* | *0.450* |
|  |  | T/T | 1 | 8 | 13 |  |  |  |  |  |
| *ABCB1* | rs1045642 | C/C | 0 | 8 | 13 |  |  | 0.325 | 0.561 | 0.259 |
|  |  | C/T | 2 | 3 | 13 |  |  | *0.500* | *0.325* | *1.000* |
|  |  | T/T | 1 | 5 | 6 |  |  |  |  |  |
| *ABCB1* | rs2032582 | A/A+A/G+G/G | 1 | 7 | 8 |  |  | 0.750 | 0.449 | 1.000 |
|  |  | A/T+G/T | 1 | 4 | 21 |  |  | ***0.017**** | *0.062* | *0.228* |
|  |  | T/T | 1 | 5 | 3 |  |  |  |  |  |
| *ABCG2* | rs2231142 | C/C | 2 | 8 | 12 |  |  | 0.100 | 0.319 | 0.571 |
|  |  | A/C | 0 | 7 | 16 |  |  | *0.560* | *1.000* | *0.383* |
|  |  | A/A | 1 | 1 | 4 |  |  |  |  |  |
| *ABCG2* | rs2622604 | C/C | 3 | 12 | 26 |  |  | 1.000 | 1.000 | 1.000 |
|  |  | C/T | 0 | 4 | 4 |  |  | *0.513* | *0.523* | *1.000* |
|  |  | T/T | 0 | 0 | 2 |  |  |  |  |  |
| *ABCG2* | rs2231137 | G/G | 3 | 11 | 18 |  |  | 0.619 | 1.000 | 0.285 |
|  |  | G/A | 0 | 4 | 12 |  |  | *0.467* | *1.000* | *0.247* |
|  |  | A/A | 0 | 1 | 2 |  |  |  |  |  |
| *POR* | rs17685 | G/G | 1 | 7 | 12 |  |  | 0.516 | 0.364 | 1.000 |
|  |  | G/A | 1 | 7 | 16 |  |  | *0.855* | *1.000* | *0.774* |
|  |  | A/A | 1 | 2 | 4 |  |  |  |  |  |
| *POR* | rs1057868 | C/C | 1 | 5 | 11 |  |  | 0.755 | 0.449 | 1.000 |
|  |  | C/T | 1 | 9 | 15 |  |  | *1.000* | *1.000* | *1.000* |
|  |  | T/T | 1 | 2 | 6 |  |  |  |  |  |
| *EGFR* | rs2293348 | C/C | 3 | 14 | 26 |  |  | 1.000 |  |  |
|  |  | C/T | 0 | 2 | 6 |  |  | *0.694* |  |  |
|  |  |  |  |  |  |  |  |  |  |  |
| *EGFR* | rs4947492 | G/G | 1 | 1 | 5 |  |  | 0.512 | 1.000 | 0.364 |
|  |  | G/A | 1 | 8 | 13 |  |  | *0.926* | *1.000* | *0.699* |
|  |  | A/A | 1 | 7 | 14 |  |  |  |  |  |
| *EGFR* | rs11977388 | T/T | 1 | 10 | 19 |  |  | 0.225 | 1.000 | 0.561 |
|  |  | T/C | 2 | 3 | 9 |  |  | *1.000* | *1.000* | *1.000* |
|  |  | C/C | 0 | 3 | 4 |  |  |  |  |  |
| *EGFR* | rs2227983 | G/G | 0 | 3 | 4 |  |  | 0.719 | 0.610 | 1.000 |
|  |  | G/A | 2 | 5 | 11 |  |  | *0.927* | *0.776* | *1.000* |
|  |  | A/A | 1 | 8 | 17 |  |  |  |  |  |
| *EGFR* | rs884225 | T/T | 1 | 4 | 10 |  |  | 1.000 | 1.000 | 1.000 |
|  |  | T/C | 2 | 9 | 16 |  |  | *0.927* | *1.000* | *0.761* |
|  |  | C/C | 0 | 3 | 6 |  |  |  |  |  |

^a^First row, grade 0 versus grade ≥1 (Fisher’s exact test); second row (italicized), grade ≤1 versus grade ≥2 (Fisher’s exact test).

^b^Any skin disorder, including skin rash, dry skin, pruritus, and paronychia.

** p* < 0.05, considered statistically significant (bold).

**Table F3.** Association between germline polymorphisms and decreased lymphocyte count level

|  |  |  | Decreased lymphocyte count level | | | | | | | |
| --- | --- | --- | --- | --- | --- | --- | --- | --- | --- | --- |
|  |  |  | Grade (No. of patients) | | |  |  |  | *p*-value^a^ |  |
| Gene | SNP ID | Genotype | 0 | 1 | 2+ |  |  | Additive  model | Recessive model | Dominant  model |
| *ABCB1* | rs1128503 | C/C | 6 | 2 | 0 |  |  | 0.794 | 0.565 | 0.694 |
|  |  | C/T | 13 | 4 | 3 |  |  | *0.132* | *0.084* | *0.184* |
|  |  | T/T | 13 | 2 | 7 |  |  |  |  |  |
| *ABCB1* | rs1045642 | C/C | 16 | 3 | 1 |  |  | 0.129 | 0.735 | 0.074 |
|  |  | C/T | 9 | 4 | 5 |  |  | *0.071* | *0.225* | ***0.037**** |
|  |  | T/T | 7 | 1 | 4 |  |  |  |  |  |
| *ABCB1* | rs2032582 | A/A+A/G+G/G | 10 | 5 | 0 |  |  | 0.853 | 0.705 | 1.000 |
|  |  | A/T+G/T | 17 | 2 | 7 |  |  | ***0.033**** | *0.358* | ***0.022**** |
|  |  | T/T | 5 | 1 | 3 |  |  |  |  |  |
| *ABCG2* | rs2231142 | C/C | 14 | 4 | 3 |  |  | 0.456 | 0.399 | 0.774 |
|  |  | A/C | 13 | 4 | 6 |  |  | *0.777* | *1.000* | *0.488* |
|  |  | A/A | 5 | 0 | 1 |  |  |  |  |  |
| *ABCG2* | rs2622604 | C/C | 26 | 5 | 10 |  |  | 1.000 | 1.000 | 1.000 |
|  |  | C/T | 5 | 2 | 0 |  |  | *0.436* | *1.000* | *0.174* |
|  |  | T/T | 1 | 1 | 0 |  |  |  |  |  |
| *ABCG2* | rs2231137 | G/G | 23 | 3 | 6 |  |  | 0.225 | 1.000 | 0.139 |
|  |  | G/A | 7 | 4 | 4 |  |  | *0.851* | *1.000* | *1.000* |
|  |  | A/A | 2 | 1 | 0 |  |  |  |  |  |
| *POR* | rs17685 | G/G | 11 | 4 | 5 |  |  | 0.345 | 0.398 | 0.370 |
|  |  | G/A | 15 | 3 | 5 |  |  | *0.495* | *0.319* | *0.494* |
|  |  | A/A | 6 | 1 | 0 |  |  |  |  |  |
| *POR* | rs1057868 | C/C | 9 | 4 | 4 |  |  | 0.536 | 1.000 | 0.352 |
|  |  | C/T | 17 | 2 | 5 |  |  | *0.899* | *0.665* | *0.717* |
|  |  | T/T | 6 | 2 | 1 |  |  |  |  |  |
| *EGFR* | rs2293348 | C/C | 28 | 6 | 8 |  |  | 0.436 |  |  |
|  |  | C/T | 4 | 2 | 2 |  |  | *0.653* |  |  |
|  |  |  |  |  |  |  |  |  |  |  |
| *EGFR* | rs4947492 | G/G | 5 | 2 | 0 |  |  | 0.565 | 0.249 | 1.000 |
|  |  | G/A | 15 | 2 | 4 |  |  | *0.389* | *0.302* | *0.319* |
|  |  | A/A | 12 | 4 | 6 |  |  |  |  |  |
| *EGFR* | rs11977388 | T/T | 18 | 3 | 8 |  |  | 1.000 | 1.000 | 0.774 |
|  |  | T/C | 9 | 3 | 2 |  |  | *0.292* | *0.319* | *0.160* |
|  |  | C/C | 5 | 2 | 0 |  |  |  |  |  |
| *EGFR* | rs2227983 | G/G | 5 | 2 | 0 |  |  | 0.604 | 0.377 | 1.000 |
|  |  | G/A | 13 | 3 | 2 |  |  | *0.112* | *0.074* | *0.319* |
|  |  | A/A | 14 | 3 | 8 |  |  |  |  |  |
| *EGFR* | rs884225 | T/T | 10 | 2 | 3 |  |  | 0.533 | 0.459 | 1.000 |
|  |  | T/C | 15 | 5 | 6 |  |  | *0.895* | *0.665* | *1.000* |
|  |  | C/C | 7 | 1 | 1 |  |  |  |  |  |

^a^First row, grade 0 versus grade ≥1 (Fisher’s exact test); second row (italicized), grade ≤1 versus grade ≥2 (Fisher’s exact test).

** p* < 0.05, considered statistically significant (bold).

*n* = 50 (data successfully collected from 50 patients)
